# Structural Insight into Protective Alumina Coatings for Layered Li-Ion Cathode Materials by Solid-State NMR Spectroscopy

**DOI:** 10.1021/acsami.3c16621

**Published:** 2024-02-02

**Authors:** Abby R. Haworth, Beth I. J. Johnston, Laura Wheatcroft, Sarah L. McKinney, Nuria Tapia-Ruiz, Sam G. Booth, Alisyn J. Nedoma, Serena A. Cussen, John M. Griffin

**Affiliations:** † Department of Chemistry, Lancaster University, Lancaster LA1 4YB, U.K.; ‡ Department of Materials Science and Engineering, University of Sheffield, Sheffield S1 3JD, U.K.; § Department of Chemistry, Molecular Sciences Research Hub, White City Campus, Imperial College London, London W12 0BZ, U.K.; ∥ Department of Chemical and Biological Engineering, University of Sheffield, Sheffield S1 3JD, U.K.; ⊥ The Faraday Institution, Quad One, Harwell Campus, Didcot OX11 0RA, U.K.

**Keywords:** Li-ion batteries, protective coatings, layered cathodes, Ni-rich cathodes, local structure, solid-state NMR spectroscopy

## Abstract

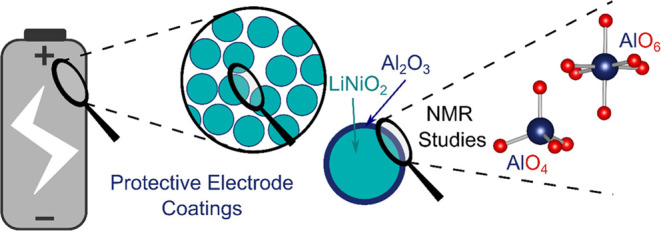

Layered transition metal oxide cathode materials can exhibit high energy densities in Li-ion batteries, in particular, those with high Ni contents such as LiNiO_2_. However, the stability of these Ni-rich materials often decreases with increased nickel content, leading to capacity fade and a decrease in the resulting electrochemical performance. Thin alumina coatings have the potential to improve the longevity of LiNiO_2_ cathodes by providing a protective interface to stabilize the cathode surface. The structures of alumina coatings and the chemistry of the coating–cathode interface are not fully understood and remain the subject of investigation. Greater structural understanding could help to minimize excess coating, maximize conductive pathways, and maintain high capacity and rate capability while improving capacity retention. Here, solid-state nuclear magnetic resonance (NMR) spectroscopy, paired with powder X-ray diffraction and electron microscopy, is used to provide insight into the structures of the Al_2_O_3_ coatings on LiNiO_2_. To do this, we performed a systematic study as a function of coating thickness and used LiCoO_2_, a diamagnetic model, and the material of interest, LiNiO_2_. ^27^Al magic-angle spinning (MAS) NMR spectra acquired for thick 10 wt % coatings on LiCoO_2_ and LiNiO_2_ suggest that in both cases, the coatings consist of disordered four- and six-coordinate Al–O environments. However, ^27^Al MAS NMR spectra acquired for thinner 0.2 wt % coatings on LiCoO_2_ identify additional phases believed to be LiCo_1–*x*_Al_*x*_O_2_ and LiAlO_2_ at the coating–cathode interface. ^6,7^Li MAS NMR and *T*_1_ measurements suggest that similar mixing takes place near the interface for Al_2_O_3_ on LiNiO_2_. Furthermore, reproducibility studies have been undertaken to investigate the effect of the coating method on the local structure, as well as the role of the substrate.

## 1 Introduction

Lithium (Li)-ion batteries are playing an important role in the transition toward a sustainable future. In particular, Li-ion batteries are widely used in portable electronic devices and hybrid and electric vehicles, and are gaining interest as potential energy storage solutions for renewable energy sources. However, there are still challenges that need to be overcome, e.g., improving energy density and cycle life.^1−3^ Recently, cathode materials with high nickel contents have attracted interest for their high energy densities.^3,4^ For layered transition metal oxides, as the nickel content is increased, from LiCoO_2_ (LCO) through LiNi_*x*_Mn_*y*_Co_1–*x*–*y*_O_2_ (NMC) to LiNiO_2_ (LNO), an increase in energy density is observed as a result of increasing the degree of (de)lithiation achieved during cycling and thus leading to higher reversible specific capacities at practical operating voltages. Indeed, LNO has gained considerable recent attention as it has the highest specific capacity of the series within a stable electrochemical window.^5−8^ However, LNO can be challenging to synthesize and suffers from significant capacity fade over a number of charge–discharge cycles. There are several compounding factors that contribute to this capacity fade, including phase changes and degradation due to unwanted reactions occurring at the cathode–electrolyte interface.^9^

Protective coatings, such as ZrO_2_, TiO_2_, and Al_2_O_3_,^10^ can be used to improve the electrode longevity and increase the lifetime of the battery. Al_2_O_3_ coatings are particularly noteworthy due to their low cost, compatibility with a wide range of systems, and ability to be coated onto cathodes using a range of techniques, including wet chemical methods and atomic layer deposition.^11^ The layer of chemically stable Al_2_O_3_ protects the cathode from unfavorable reactions occurring between the active material and the electrolyte during electrochemical cycling and has been reported to react with and/or scavenge HF.^12,13^ As such, alumina coatings have been reported to improve the capacity retention of a range of electrodes including LCO, NMC, and LiMn_2_O_4_ (LMO).^13−15^ For Al_2_O_3_ coatings on LCO, an optimal mass loading of 0.2 wt % resulted in a capacity retention of over 95% after 50 cycles.^16^ Additionally, in some cases, Al_2_O_3_ coatings have been shown to improve cell safety and increase rate capability.^12^

In order to optimize these coatings, it is important to understand the role their structure plays in their performance as a protective coating. At optimal loadings of around 0.2 wt %, the coatings are very thin and yield low sensitivity for many characterization techniques. The structure is largely amorphous as a result of the sol–gel synthesis, which limits the utility of many diffraction-based structure characterization methods. Furthermore, the coating–cathode interface, which is of particular interest, is buried within the system, making it more challenging to study via traditional surface-sensitive techniques.

When first reported as a coating for LCO, it was noted that the powder X-ray diffraction (PXRD) data showed no indication of a crystalline Al_2_O_3_ phase.^14^ This is commonly observed for thin oxide coatings due to the lack of long-range order and the thin nature of the coatings. However, rather than a distinct coating, Auger electron spectroscopy (AES) and XRD suggested the presence of LiCo_1–*x*_Al_*x*_O_2_ and a compositional gradient (0 < *x* < 0.5) at the surface. When compared to Al-doped LCO and uncoated LCO, Al_2_O_3_-coated LCO showed significantly improved electrochemical performance.^14^ Despite the observed improvement in the performance, the precise structure of this coating remained unclear.

Since the initial reports, there have been a number of studies on alumina-coated cathode materials. Electron microscopy paired with electron-dispersive X-ray (EDX) analysis has been used to map where elements such as Al are located within the sample,^13,17−19^ while X-ray photoelectron spectroscopy (XPS) provides information about the chemical and bonding environments.^15,18,20^ However, the information provided by these surface-sensitive techniques may not be representative of the bulk and cannot provide insight into the buried interfaces. If a cross section is taken through the sample, it is possible to examine the interfaces via electron microscopy and EDX. However, this process can introduce contamination. Hence, the structures of Al_2_O_3_ coatings and the nature of the coating–cathode interface remain underexplored and are still debated.

Solid-state NMR spectroscopy is a versatile technique that has no requirement for long-range order, making it ideally suited to the study of disordered systems, such as coatings and interfaces.^21,22^ The relationship between the ^27^Al chemical shift and the Al coordination environment is well reported in the literature.^23^ Typically, solid-state NMR studies for coatings on LCO suggest that the coating is made up of four- and/or six-coordinate Al environments.^20,24−27^ Additionally, a number of studies indicate the presence of a LiCo_1–*x*_Al_*x*_O_2_ phase, in agreement with the initial study.^20,25,27^ For Al_2_O_3_ coatings on NMC, the reported structure is dependent on the NMC compositions. For coatings of Al_2_O_3_ on NMC532 (LiNi_0.5_Mn_0.3_Co_0.2_O_2_), a LiAlO_2_ phase was identified as forming at the coating–cathode interface.^27^ However, changing the composition from NMC532 to NMC662 (LiNi_0.6_Mn_0.2_Co_0.2_O_2_) and NMC811 (LiNi_0.8_Mn_0.1_Co_0.1_O_2_) enabled the diffusion of Al into the bulk of the cathode in addition to the formation of a LiAlO_2_/Al_2_O_3_ coating.^28,29^

In this work, we use solid-state NMR spectroscopy to study the structure of Al_2_O_3_ coatings on LCO and LNO. The main focus of this work is on the structural characterization of Al_2_O_3_ coatings, although electrochemical data is presented to confirm that a coating enhances capacity retention in LNO as compared to an uncoated cathode. We perform a systematic structural study as a function of alumina mass loading and use LCO as a model substrate to guide our interpretation of the results acquired for the more challenging paramagnetic LNO. Although the impact on the chemical shift range observed in the NMR spectrum could help provide information about species in the coating versus dopants in bulk LNO, this paramagnetic interaction often also results in fast relaxation times and broadened resonances. Paired with the small sample volume and disorder present in these coatings, the paramagnetic nature of LNO has the potential to make acquiring and interpreting NMR data more challenging. It is noted that in previous studies of NMC systems reported, the transition metals present can affect the coating–cathode interface; however, Han et al. suggest that it is the Mn content of the NMC systems that result in the differences observed.^28^ Thus, by utilizing variations in coating thickness, and combined LCO and LNO analysis, it is possible to study the structure of Al_2_O_3_ coatings and gain insight allowing for future coating optimization to preserve the high capacity and rate capability of the material.

## 2 Experimental Section

### 2.1 Synthesis

LiNiO_2_ (synthesized via the method below), LiCoO_2_ (Sigma-Aldrich—99.8%), and MgO (Alfa Aesar—99.96%) were coated in 10, 2, and/or 0.2 wt % coatings of Al_2_O_3_ via a solution method. LiNiO_2_ was synthesized by mixing together Ni(OH)_2_ and LiOH·H_2_O powders together using a mortar and pestle. The mixture was transferred to a tube furnace and preannealed at 480 °C for 5 h followed by a final annealing step at 710 °C for 15 h, with heating rates of 5 °C min^–1^. Both steps were carried out under a continuous flow of O_2_. After annealing, samples were held at 200 °C before transfer to an Ar-filled glovebox to minimize air exposure and moisture uptake to the substrate before coating. To create a coated particle, the substrate is added to a solution of Al(NO_3_)_3_.9H_2_O (Alfa Aesar) in ethanol. Solution concentrations ranged from 1 to 20 mg mL^–1^ depending on the required coating weight and the need to produce a volume of solution that could be easily handled. The desired mass of Al_2_O_3_ (thus volume of solution needed) was calculated from the mass of substrate used in the coating process: i.e., for the 10 wt % coating, if using 100 mg of LiNiO_2_, then 10 mg of Al_2_O_3_ is required with the appropriate volume of solution calculated from the solution concentration. While stirring, it is then heated to 50–60 °C to gently evaporate the ethanol. The resulting gel/powder is heated at 400 °C for 3 h in air to form a Al_2_O_3_ coating. For a 0 wt % coating control, the same procedure was carried out using pure ethanol rather than a solution of Al(NO_3_)_3_.9H_2_O in ethanol. To create a control sample of Al_2_O_3_, the coating procedure was carried out without the addition of a substrate to the precursor solution.

### 2.2 Powder X-ray Diffraction

Powder X-ray diffraction (PXRD) data was acquired using a Rigaku Miniflex diffractometer in reflection mode using Cu Kα (λ = 1.5406 Å) radiation. Data was acquired from 2θ = 10 to 90° with a step size of 0.02°.

### 2.3 Electron Microscopy

#### 2.3.1 LiNiO_2_

Powder samples were sonicated for 5 min in ethanol before being drop-cast onto lacy carbon Cu TEM grids (EMR). These were then transferred to a JEOL JEM F200 transmission electron microscope (TEM). Transmission electron microscopy (TEM) images were acquired at 200 kV using a Gatan OneView Camera, while Gatan high-angle annular dark-field (HAADF) and bright-field (BF) detectors were used to acquire HAADF and BF scanning transmission electron microscopy (STEM) images, respectively.

#### 2.3.2 LiCoO_2_

Powder samples were sonicated for 5 min in acetone before being drop-cast onto holey carbon Cu TEM grids (EMR). These were then transferred to a JEOL STEM 2100Plus transmission electron microscope. Transmission electron microscopy (TEM) images were acquired at 200 kV by using a Gatan Orius Camera.

### 2.4 Electrochemical Characterization

LiNiO_2_ powders were mixed with C65 conductive carbon (Imerys) and poly(vinylidene fluoride) (PVDF) (Aldrich) in a weight ratio of 90:5:5 before being dispersed in *N*-methyl-2-pyrrolidone (NMP) solvent (Merck) and mixed in a planetary mixer to obtain a slurry. The slurry was cast onto carbon-coated Al foil using a doctor blade set at a height of 150 μm. The slurry was dried at 100 °C to evaporate the NMP before further drying at 80 °C overnight in a vacuum oven. Electrodes of 10 mm diameter were punched from the dried film and transferred to an Ar-filled glovebox. Coin cells (CR2023 type) were constructed using the punched LiNiO_2_ disks as cathode, a 15.8 mm diameter Li metal anode (Cambridge Energy Solutions), and a Whatman glass microfibre separator. 100 μL of 1 M LiPF_6_ in ethylene carbonate (EC):ethyl methyl carbonate (EMC) (EC:EMC 3:7 by weight) with 2% vinylene carbonate (VC) additive (Solvionic) was used as electrolyte.

Galvanostatic charge–discharge tests were carried out in a temperature-controlled chamber (25 °C) connected to an Arbin LBT21084 cycler. For both cells, two formation cycles were initially carried out at a rate of C/20 (where 1C was defined as 220 mA g^–1^) between 3.0 and 4.3 V vs Li/Li^+^. The cycling rate was then increased to C/3 for 100 cycles with the same voltage window as those for previous cycles.

### 2.5 Solid-State NMR Spectroscopy

Solid-state NMR data was acquired using a Bruker Avance Neo spectrometer equipped with a 23.5 T standard-bore magnet or a Bruker Avance III HD spectrometer equipped with either a 16.4 or 9.4 T wide-bore magnet. Larmor frequencies of 260.6 and 182.4 MHz were used for ^27^Al (*I* = 5/2) at 23.5 and 16.4 T, respectively. Larmor frequencies of 58.9 and 155.5 MHz were used for ^6^Li (*I* = 1) and ^7^Li (*I* = 3/2) at 9.4 T, respectively. Powdered samples were packed into conventional 1.9, 2.5, and 3.2 mm ZrO_2_ rotors, and magic-angle spinning (MAS) rates between 40 and 18 kHz were employed. ^27^Al chemical shifts were referenced to Al(NO_3_)_3_ (aq) using Al(acac)_3_ (s) as a secondary reference (δ_iso_ = −0.13 ppm, *C*_Q_ = 2.99 MHz, and η_Q_ = 0.16), while ^6,7^Li chemical shifts were referenced to 1 M LiCl (aq) using a secondary reference of Li_2_CO_3_ (s) (δ = 0.11 ppm).

^27^Al MAS NMR spectra were acquired using a single-pulse experiment with typical pulse lengths of 2.75 and 1.5 μs at 23.5 and 16.4 T, respectively, and an experimentally optimized recycle interval of 0.5 s was used. For spectra acquired using a Hahn echo experiment (90-τ-180) at 23.5 T, a low power π/2 pulse length of 30 μs was used. ^27^Al cross polarization (CP) MAS NMR spectra were acquired using CP from ^1^H using a contact pulse duration of 100 μs (ramped for ^1^H). Two-pulse phase modulation (TTPM) ^1^H decoupling was then applied during acquisition. At 16.4 T, ^27^Al multiple-quantum (MQ)MAS NMR experiments were acquired using a phase-modulated split-*t*_1_ pulse sequence with whole-echo acquisition.^30^^7^Li MAS NMR spectra were acquired at 9.4 T using conventional single-pulse experiments with a pulse length of 3 μs. ^6^Li MAS NMR spectra were acquired by using a Hahn echo experiment with 90 and 180° pulse lengths of 3 and 6 μs, respectively. Additional experiment-specific parameters are available in the relevant figure caption.

## 3 Results and Discussion

### 3.1 Initial Characterization

**Figure 1:**
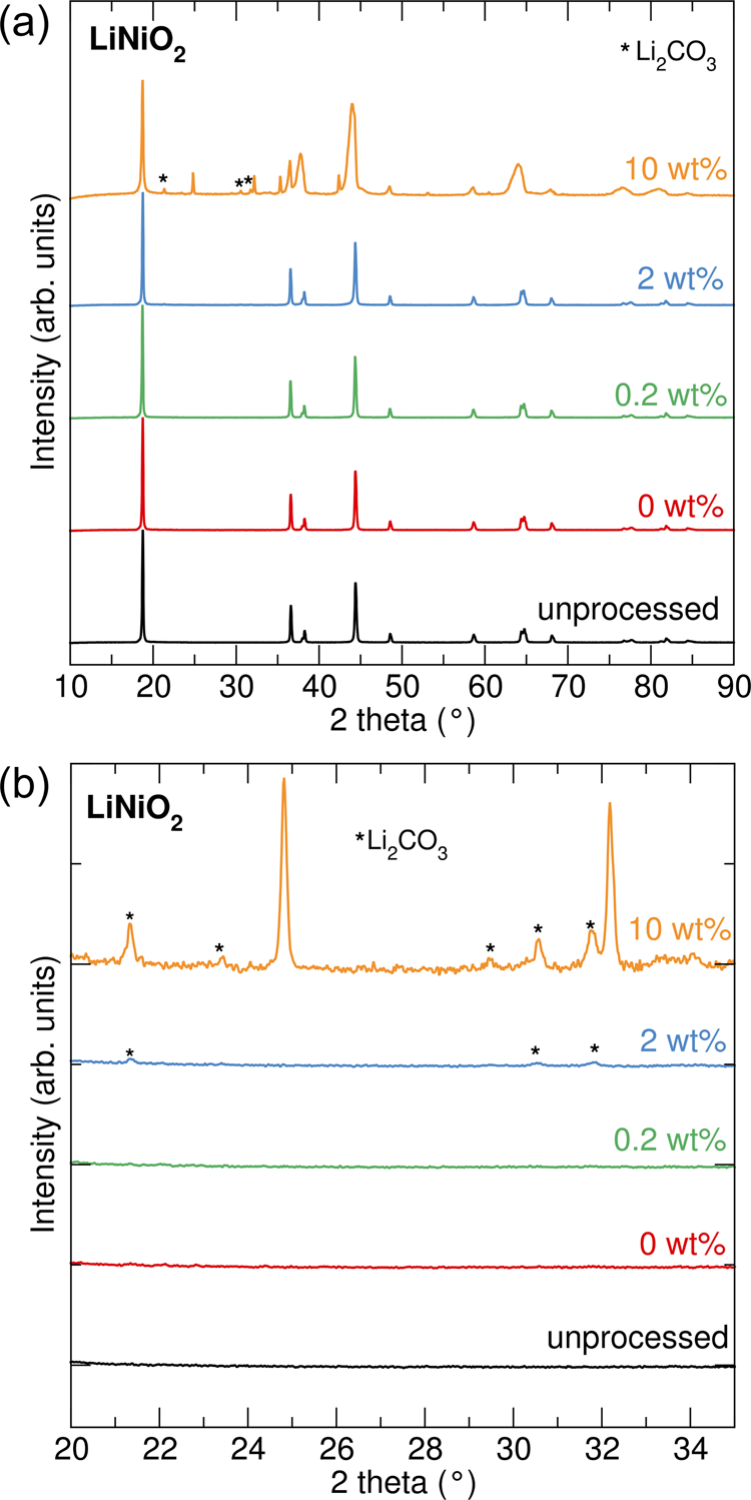
(a, b) Powder X-ray diffraction patterns for unprocessed LiNiO_2_ and 0, 0.2, 2, and 10 wt % coatings of Al_2_O_3_ on LiNiO_2_. Additional Li_2_CO_3_ phases are highlighted (*). Part (b) shows an expansion between 20 and 34° 2θ of (a).

Al_2_O_3_ coatings were deposited via a solution method, as described in the Experimental Section. PXRD data was obtained for unprocessed LNO as well as 0.2, 2, and 10 wt % coatings of Al_2_O_3_ on LNO and LCO, as shown in Figure 1 (LNO) and Figure S2 (LCO). 0.2 wt % is representative of typical coatings used in battery cells; 2 and 10 wt % were also studied here for comparison to provide additional structural insight. Data were also obtained for an LNO sample that was subjected to the same solution process but without the addition of Al(NO_3_)_3_.9H_2_O (hereafter referred to as 0 wt %). Comparison of the unprocessed LNO with the 0 wt % coated LNO suggests that LNO is unaffected by the coating procedure as no change in the PXRD pattern is observed. For both the 0.2 and 2 wt % coatings of Al_2_O_3_ on LNO, no additional Al_2_O_3_ phases are observed in the PXRD pattern. This suggests that the coating is disordered and too thin to observe. A PXRD pattern of pure Al_2_O_3_ prepared by the same solution method (Figure S14) also indicates an amorphous structure. It is noted that for the 2 wt % Al_2_O_3_ coating, additional very low-intensity reflections consistent with Li_2_CO_3_ (indicated with * in Figure 1b) are observed. Indeed, Ni-rich layered oxides, especially LNO, are reported to be particularly susceptible to residual lithium species, such as Li_2_CO_3_.^31,32^ However, the PXRD pattern for the 10 wt % Al_2_O_3_ coating on LNO indicates the presence of multiple additional phases alongside broadening of peaks with *hk* character which can happen due to strain in the *ab* plane. The interlayer spacing appears unaffected, as evidenced by the (003) and (006) peaks not undergoing the same broadening. In addition to Li_2_CO_3_ (indicated with * in Figure 1b), there is at least one unidentified phase. It is likely that due to the sensitive surface chemistry of LNO, the coating process with a large amount of Al_2_O_3_ leads to a degree of surface reconstruction where Li leaching from LNO can occur that can act to partially delithiate LNO near the surface and lead to the formation of Li-containing phases at the Al_2_O_3_-LNO interface. This phase could be close in composition to LiAl_5_O_8_ or crystalline α-Al_2_O_3_ (corundum, Figure S15), although differences in peak positions compared to reference patterns obtained (ICSD 66941 for Al_2_O_3_, 10,480 for LiAl_5_O_8_) indicate some deviation in lattice parameters that could be explained by partially lithiated species instead of ideal stoichiometry. For LCO, the PXRD patterns of all coatings (0.2, 2, and 10 wt %) of Al_2_O_3_ show no significant differences as compared to the uncoated sample. The differences in behavior observed for the 10 wt % coatings on LNO and LCO are most likely due to the increased surface instability associated with LNO compared to LCO.

**Figure 2:**
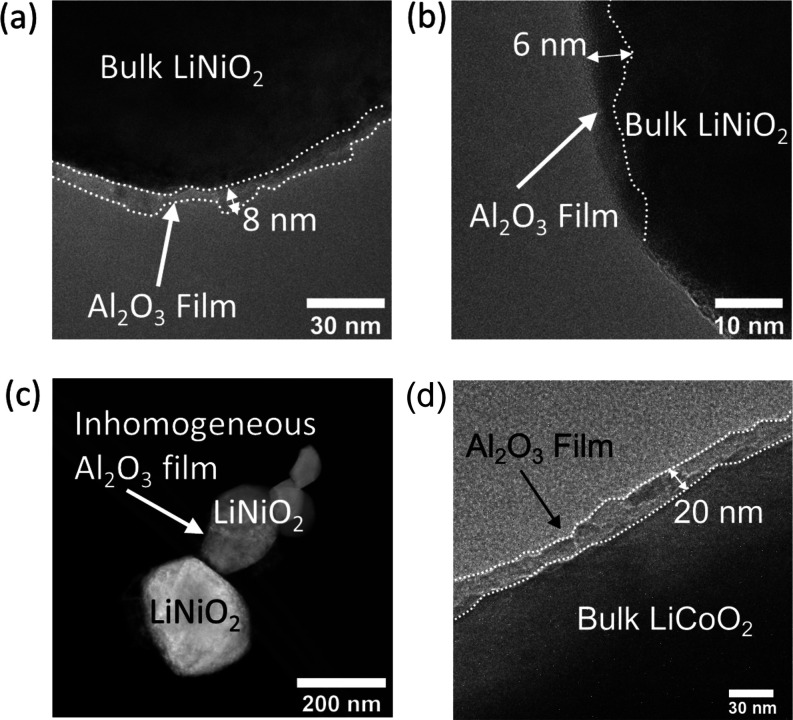
(a, b) TEM images of the 2 wt % Al_2_O_3_ on LiNiO_2_ showing the 6–8 nm thick film. (c) High-angle annular dark-field scanning transmission electron microscopy (HAADF-STEM) image of Al_2_O_3_ coated LiNiO_2_ showing the inhomogeneous nature of the Al_2_O_3_ coating. (d) TEM image of the 2 wt % Al_2_O_3_ on LiCoO_2_ showing a 20 nm thick film.

In order to confirm the amorphous nature of the coating and to investigate the thickness and distribution, electron microscopy studies were carried out for the 2 wt % coating. Figure 2 shows the TEM images collected for a particle of LNO coated in 2 wt % Al_2_O_3_. The TEM images obtained show an amorphous coating of 6–8 nm on crystalline particles, suggesting that the Al_2_O_3_ coats the LNO. For the 2 wt % coating on LCO, an amorphous coating of 20 nm is observed. While no separate amorphous particles were observed, some thicker, bunched regions of coating were observed by TEM. This is in agreement with Al_2_O_3_ coatings on LCO/NMC reported in the literature.^13,16,27,28^ Furthermore, it is noted that Al_2_O_3_ does not coat the entire particle surface, which is expected for coatings with low mass loading.

### 3.2 Electrochemical Characterization

**Figure 3:**
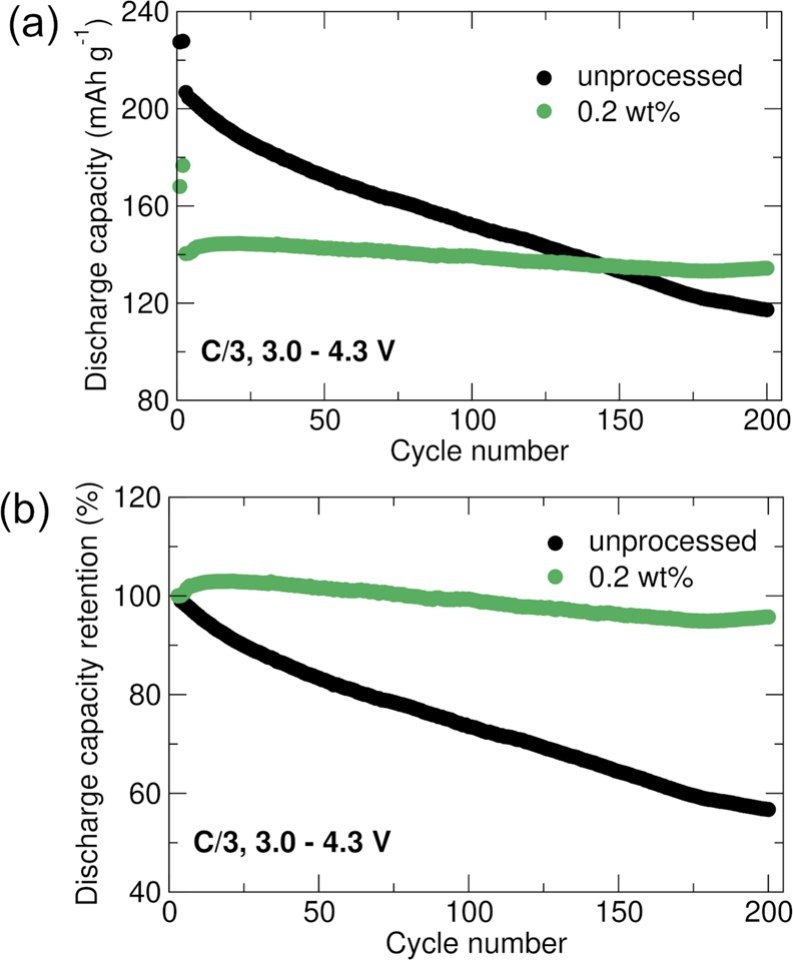
Comparison of (a) specific discharge capacity stability and (b) normalized capacity retention for uncoated LiNiO_2_ and LiNiO_2_ with 0.2 wt % Al_2_O_3_ coating obtained from galvanostatic cycling of half coin cells between 3.0 and 4.3 V.

Galvanostatic cycling measurements were conducted on half-cells with uncoated LNO and 0.2 wt % Al_2_O_3_-coated LNO to examine the influence of the alumina coating on the electrochemical behavior. Clear changes are observed in the electrochemistry upon coating. The charge–discharge profiles for cycle 2 (after the first conditioning cycle) are shown in Figure S1. While the typical plateaus associated with (de)lithiation of LNO (as a result of phase transitions) are observed across both samples, a clear drop in specific capacity at C/20 is noted for coated LNO (∼177 mAh g^–1^) compared to uncoated LNO (∼228 mAh g^–1^). An increase in polarization is also evident from the higher voltages upon charge and lower voltages on discharge exhibited by the coated sample compared to the uncoated one. This suggests that the coating layer may increase the Li^+^ charge transfer resistance at the surface. Furthermore, the possibility of LiNi_1–*x*_Al_*x*_O_2_ phases at the Al_2_O_3_–LiNiO_2_ interface is likely to decrease the capacities as a result of the presence of electrochemically inactive Al^3+^. Similar observations have been reported on Al_2_O_3_-coated LiNi_0.5_Mn_0.3_Co_0.2_O_2_ (NMC532), where it is suggested that possible Li loss from the bulk forms Li-containing surface species during the wet coating process.^27^ Such Li leaching from the electrochemically active cathode structure compounded with the formation of resistive surface species leads to the capacity drop on coating. We suspect that the more severe capacity drop we observe here is attributed to the much increased surface sensitivity of LiNiO_2_ compared to NMC532, where surface reconstructions are likely to occur to a much greater extent.^42^ However, an improvement in cycling stability is clearly highlighted in Figure 3a, with the coated sample exhibiting stable capacities and losses of only ∼10 mAh g^–1^ after 200 cycles at C/3. For comparison, the uncoated LNO suffers from losses of ∼90 mAh g^–1^ under the same conditions. We note that the initial capacity of the coated LNO is significantly lower than that of the uncoated LNO, suggesting that the coating process could be further optimized. However, the capacity of the uncoated electrode drops below that of the coated electrode after ∼150 cycles and continues dropping. This is further evidence that the coating protects against unwanted reactions that continue even when the number of intercalated Li ions is reduced. There is additionally a larger drop in capacity from C/20 to C/3 observed for coated LNO (∼35 mAh g^–1^) compared to uncoated LNO (∼20 mAh g^–1^), which confirms the increase in charge transfer resistance brought on by the Al_2_O_3_ coating. The superior normalized capacity retention of coated vs uncoated LNO in Figure 3b further substantiates the stability of coated LNO with 96% retention as compared to 57% retention of uncoated LNO after 200 cycles.

### 3.3 Local Structural Characterization

In order to further characterize and understand the structure of the coatings, solid-state NMR spectroscopy was carried out. As described above, due to the additional challenges introduced by paramagnetic systems, Al_2_O_3_ coatings on LCO were initially studied as a diamagnetic analogue to LNO.

**Figure 4:**
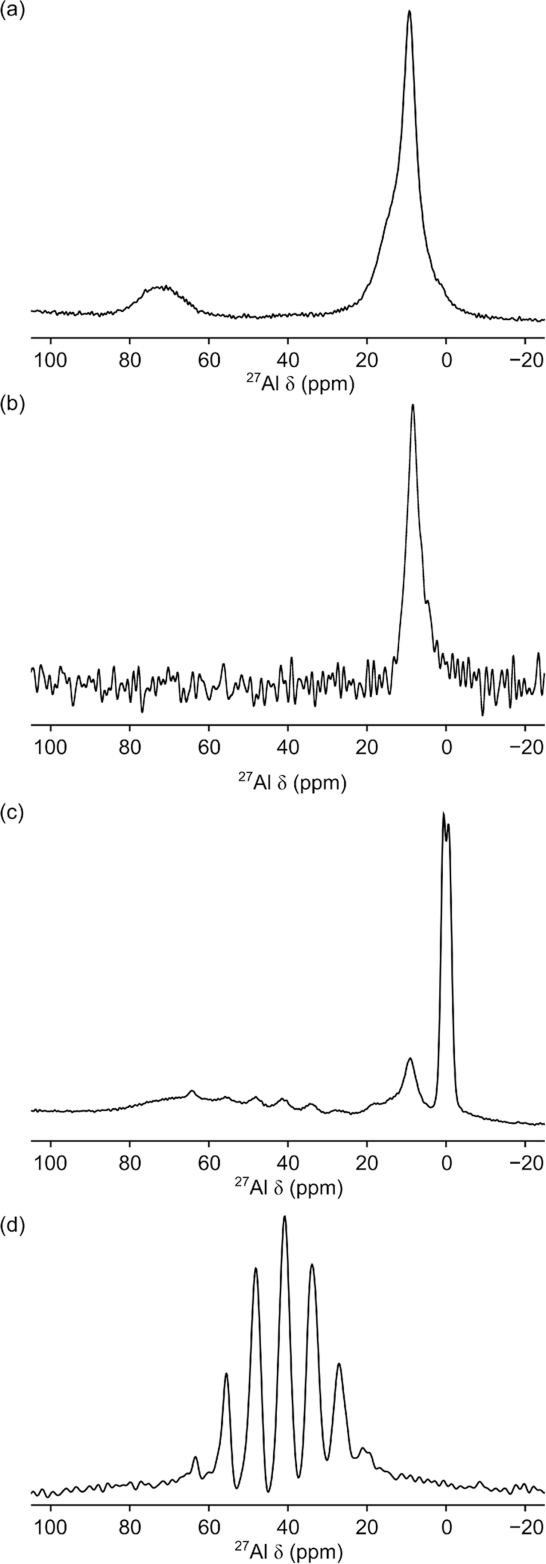
(a) ^27^Al MAS (23.5 T) NMR and (b) ^1^H–^27^Al CPMAS (16.4 T) NMR spectra of a 10 wt % coating of Al_2_O_3_ on LiCoO_2_ and ^27^Al MAS (23.5 T) NMR spectra of (c) 0.2 wt % coating of Al_2_O_3_ on LiCoO_2_, and (d) LiCo_0.5_Al_0.5_O_2_. Each spectrum is the result of averaging (a) 320, (b) 7200, (c) 29696, and (d) 320 transients with recycle intervals of (a, c, d) 0.5 or 3 s. MAS rates of (a, c, d) 40 or (b) 18 kHz were used.

The ^27^Al MAS NMR spectrum for 10 wt % Al_2_O_3_ coated on LCO is shown in Figure 4a. The spectrum exhibits two resonances at δ ≈ 73 and 9 ppm corresponding to tetrahedral and octahedral Al–O environments, respectively. This is in agreement with data previously reported for alumina coatings synthesized via wet chemical methods.^24,25,27^ The resonance observed at δ = 9 ppm does not conform to a single second-order quadrupolar-broadened line shape and is asymmetrically broadened with a shoulder at δ ≈ 13 ppm, suggesting the presence of multiple octahedral environments. The other resonance, observed at δ = 73 ppm, is broad, with no significant features. A ^27^Al MQMAS spectrum (Figure S3) also shows broad overlapping lineshapes indicating disorder. These results are consistent with the amorphous nature of the coating observed by TEM.

The loading level for the 10 wt % coating is much higher than typically used for battery cathodes, and it is possible some of the Al_2_O_3_ exists as separate particles rather than as a coating on the LCO substrate. However, when compared to the spectrum acquired for bulk Al_2_O_3_ synthesized via the coating method (Figure S4), significant differences are observed. Most notably, for the coating, no distinct resonances are observed in the five-coordinate chemical shift, although there is possibly weak intensity in this region at ∼40 ppm. This suggests that the presence of a substrate results in the formation of a local structure different from that of alumina even via the same synthetic approach. The precise influence of the substrate on the coating structure is unclear, although it is noted that Al_2_O_3_ coatings on MgO also show an absence of five-coordinate environments (Figure S5). While it is not possible to assign a single structure to the coating, the local Al environments are similar to those observed in other Al_2_O_3_ phases, such as γ-, χ-, and η-Al_2_O_3_.^23^ In particular, the spectrum is similar to that reported for α-Al_2_O_3_ in the literature.^33,34^

To further probe the structure, a ^1^H–^27^Al cross-polarization (CP) MAS NMR spectrum was recorded (Figure 4b) to identify Al species in close proximity to protons. The spectrum shows a single resonance at δ = 8 ppm corresponding to at least one six-coordinate Al environment. The lack of shouldering suggests that only some of the six-coordinate environments within the coating are in proximity to protons. While the location of the protons within the coating is unknown, we note that for other alumina phases, such as α-Al_2_O_3_, protons have been shown to be present in the form of OH groups on the surface.^33−35^ However, this does not rule out the possibility for protons to be present within the bulk of the coating structure.^36^

Figure 4c shows a ^27^Al MAS NMR spectrum for a 0.2 wt % coating on LCO. The spectrum displays a number of distinct resonances, together with broad intensity centered at approximately δ ≈ 68 ppm. Due to the similarity with resonances observed for the thicker coating and the Al_2_O_3_ synthesized via the coating method, the intensity at 68 ppm is attributed to disordered four-coordinate Al–O species within the coating. Two distinct resonances are observed in the six-coordinate Al chemical range (at δ = 0 and 9 ppm). The 0 ppm resonance is consistent with six-coordinate Al environments and assignments made for 0.5 wt % coatings on NMC532 in ref (37). The 9 ppm resonance exhibits a slight shoulder at 14 ppm. To assist in assignment, NMR data were acquired for a range of model Li–Al–O phases (Figure S7). The main candidate phase is LiAlO_2_, for which a resonance at δ = 9 ppm was observed in Figure S7. The presence of LiAlO_2_ has previously been reported in the literature for coating of Al_2_O_3_ on NMC cathodes.^27,28,37,38^ However, it was not observed previously for similarly synthesized coatings on LCO.^27^ We note that the identification of this resonance was only possible by the increased resolution at a high magnetic field (23.5 T). At lower fields, the overlap between resonances meant that it was not possible to separate the distinct environments. The shoulder at 14 ppm could be due to α-Al_2_O_3_ or another Li–Al–O phase.

Within the intermediate chemical shift range, there are at least seven additional features between δ = 64 and 18 ppm separated by approximately 7–8 ppm. These features are in good agreement with those in the spectrum acquired for LiCo_0.5_Al_0.5_O_2_ (Figure 4d) where compositional disorder results in Al environments with a variety of next-nearest neighbors, as previously reported for the LiCo_1–*x*_Al_*x*_O_2_ series by Gaudin et al.^39^ and previously observed in coatings on LCO.^27^ However, it is not possible to determine the exact Co:Al ratio for the phase present in the coating due to the low intensity of these features. The presence of the additional Al-, Co-, and Li-containing phases in the 0.2 wt % coating indicates mixing of Al, Li, and Co occurs at the coating–cathode interface during synthesis when the sample is heated to 400 °C.

These additional phases, observed in the 0.2 wt % coating, were not observed in the NMR data for the 10 wt % coating. However, it is noted that a broad bump was observed in the spectrum acquired at 23.5 T (Figure 4a) and in the spectrum (Figure S6a) acquired at 16.4 T, and broad low-intensity features are present in the chemical shift region assigned to LiCo_1–*x*_Al_*x*_O_2_. This could suggest the presence of additional phases which are masked by higher-intensity signals.

**Figure 5:**
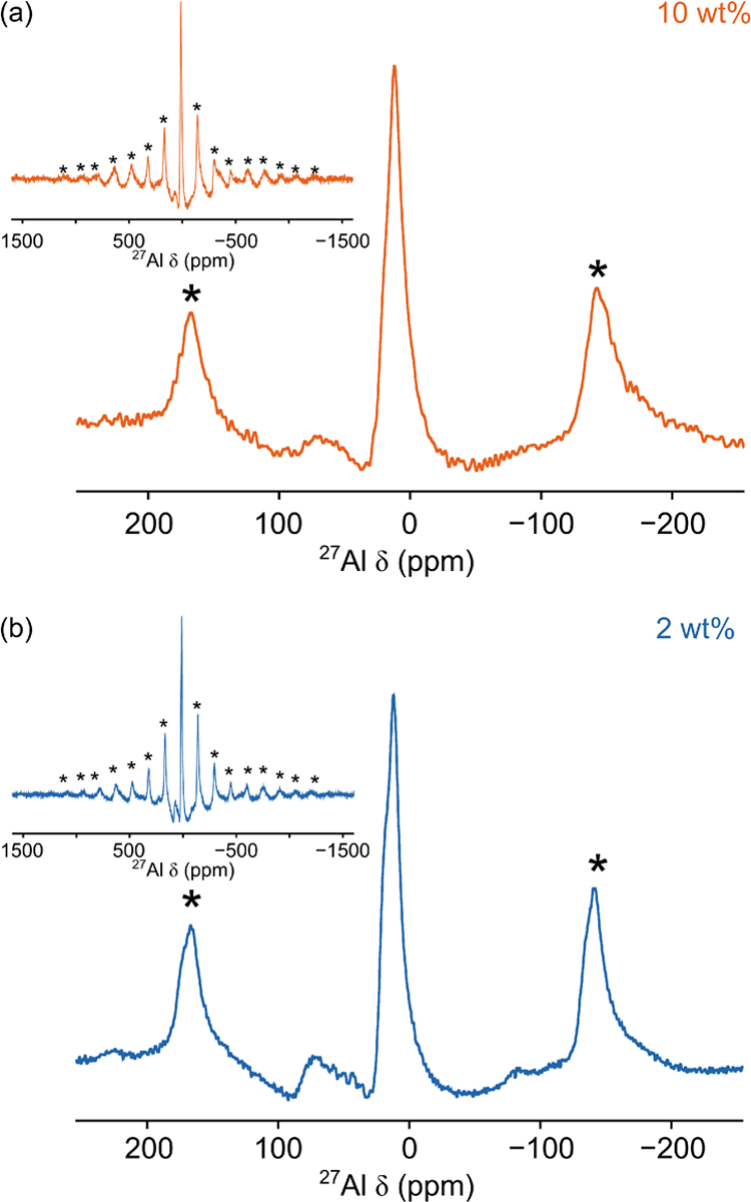
^27^Al MAS NMR (23.5 T) spectrum of (a) the 10 wt % coating of Al_2_O_3_ on LiNiO_2_ and (b) the 2 wt % coating of Al_2_O_3_ on LiNiO_2_. Each spectrum is the result of averaging (a) 960 and (b) 6400 transients with a recycle interval of 0.5 s. In both spectra, an MAS rate of 40 kHz was used.

Figure 5a shows the ^27^Al MAS NMR spectrum acquired for a 10 wt % coating of Al_2_O_3_ on LNO. The spectrum is much broader than those recorded for coatings on LCO due to the presence of paramagnetic Ni^3+^ species in the LNO substrate. Here, two broad resonances are observed centered at δ ≈ 66 and 12 ppm with a large manifold of spinning sides. These resonances are very similar to those observed for the 10 wt % coating on LCO; however, these resonances are much broader and we hypothesize these are the result of the paramagnetic interaction. The resonance at δ = 12 ppm exhibits a small shoulder at δ ≈ 18 ppm, suggesting multiple overlapping lineshapes. However, due to the broad nature of the lineshapes, it is challenging to assign individual Al environments beyond being four- and six-coordinate Al–O environments, as was the case for the 10 wt % coating on LCO. The ^27^Al MAS NMR spectrum acquired for a 2 wt % coating of Al_2_O_3_ on LNO, as for the thicker coating, exhibits two broad resonances at δ ≈ 70 and 12 ppm (Figure 5b). Again, the resonance at δ ≈ 12 ppm exhibits slight shouldering at a lower frequency, suggesting possible multiple overlapping lineshapes.

In addition to these, there are possible features between the two resonances (35 and 70 ppm) for the 2 wt % coating. However, there is a poor signal-to-noise ratio, which makes assignment challenging. It is noted that for other 2 wt % coatings on LNO, these features are not observed (see discussion below). It is possible that, based on the results observed for coatings on LCO, cation mixing may occur at the coating–cathode interface. If this were the case, we would expect to observe LiAlO_2_ and LiNi_1–*x*_Al_*x*_O_2_ phases similar to those in Figure 4c. However, it is not possible to identify any LiAlO_2_ phases as the broad line shape at δ ≈ 12 ppm overlaps with the expected chemical shift. The features observed between the two resonances exhibit a chemical shift similar to that of the ^27^Al MAS NMR spectrum acquired for LiNi_0.95_Al_0.05_O_2_ (Figure S9). However, the broadened nature of the lineshapes and the poor signal-to-noise ratio means that the features cannot be identified as signal or assigned to additional phases.

For a 0.2 wt % coating of Al_2_O_3_ on LNO, very low signal intensity was obtained even with a long experimental time of approximately 37 h and it was not possible to determine the number of resonances due to baseline distortion from pulse breakthrough (Figure S10a). Spectra acquired using a Hahn echo pulse sequence show either four- or six-coordinate environments depending on the applied magnetic field (Figure S10b,c). It is likely that the challenges in acquiring data for this sample are a result of the paramagnetic interaction with Ni^3+^ in the LNO structure. As the coating is very thin, the majority of the coating will be in close proximity to the paramagnetic LNO.

**Figure 6:**
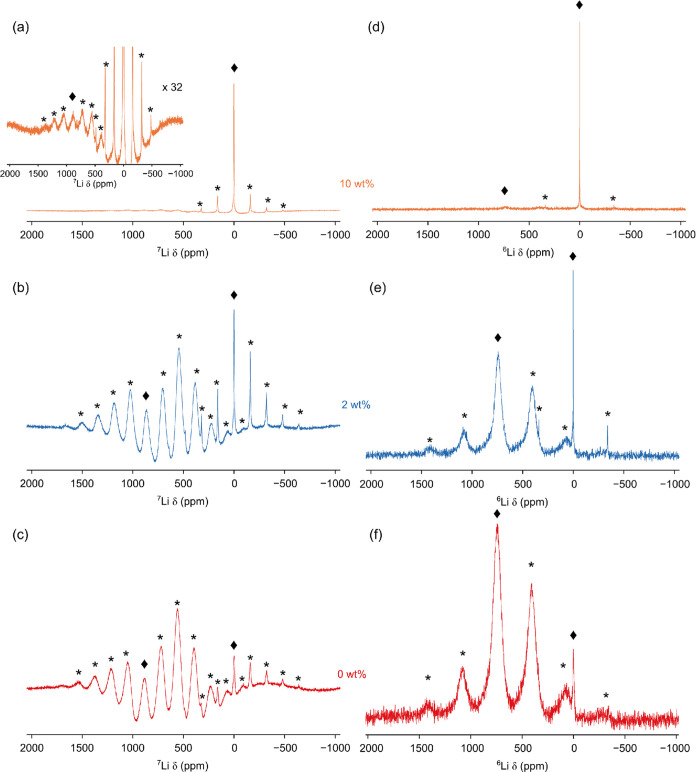
^7^Li and ^6^Li MAS (9.4 T) NMR spectra for (a, d) 10 wt %, (b, e) 2 wt %, and (c, f) 0 wt % Al_2_O_3_ coatings on LiNiO_2_. Each spectrum is the result of averaging (a, b, c) 160 or (d, e, f) 96000 transients with recycle intervals of (a, b, c) 0.5 or (d, e, f) 1 s. MAS rates of (a, b, c) 25 kHz and (d, e, f) 20 kHz were used. Spinning sidebands are denoted by *, while the isotropic resonance is denoted with a ◆.

^6,7^Li MAS NMR experiments were performed for the Al_2_O_3_-coated LNO samples (Figure 6). Typically, ^6,7^Li has a narrow chemical shift range for diamagnetic species centered around 0 ppm, which can make resolving individual Li environments challenging. However, Li^+^ ions within the LNO structure will experience a strong interaction with the paramagnetic Ni^3+^, resulting in significant broadening of the line shape and a chemical shift of δ ≈ 700 ppm.^40^ As a result, ^6/7^Li within additional (diamagnetic) phases present at the coating–cathode interface could appear as resonances either in the diamagnetic chemical shift range, e.g., LiAlO_2_, or shifted due to the incorporation of paramagnetic species, e.g., LiNi_*x*_Al_1–*x*_O_2_.

The ^7^Li MAS NMR spectrum, shown in Figure 6a–c, shows two distinct resonances exhibiting large manifolds of spinning sidebands characteristic for systems containing paramagnetic species. Thus, to obtain better resolution, ^6^Li MAS NMR spectra were also acquired. While the low natural abundance of ^6^Li (7.6%) can make it challenging to acquire data, its lower gyromagnetic ratio makes it more favorable for acquiring higher-resolution spectra in the presence of paramagnetic interactions. In the ^6^Li MAS NMR spectra (Figure 6d–f), two distinct resonances are observed at approximately δ = 742 and 0.2 ppm. The resonance at δ = 742 ppm is assigned to LNO based on its chemical shift, broad line shape, and fast relaxation.

The resonance at 0 ppm is in the chemical shift range expected for diamagnetic species and may correspond to additional phases resulting from mixing at the coating–cathode interface. However, it is noted that Li_2_CO_3_ has a similar chemical shift (δ = 0.1 ppm) and is commonly observed as an impurity in LNO samples. While PXRD data for the 0 wt % coating suggest LNO has no impurities, an additional phase is observed in the NMR data. This phase could be disordered or present in very low quantities unobserved by lab-based PXRD. It is noted that due to the long relaxation time of the diamagnetic species (see discussion below), spectra were acquired with a recycle interval optimized for the paramagnetic resonance. As a result, the experimental parameters favor the resonance assigned to LNO and, thus, the experiment is unable to directly quantify the amount of diamagnetic species present. It is still possible, however, to compare samples to each other and comment on the change in the ratio of Li in paramagnetic and diamagnetic environments as the coating mass loading increases.

The intensity of the diamagnetic resonance increases relative to that assigned to LNO as the mass loading of the coating increases. This may indicate that the thicker coatings contain more Li_2_CO_3_ or the presence of other diamagnetic lithium-containing phases formed during the coating process. It is noted that in the PXRD data of the 10 wt % coating, both Li_2_CO_3_ and another phase are observed. However, as the line shape (3.2 ppm wide at full width at half-maximum) covers a large portion of the chemical shift range of lithium, we are unable to resolve individual Li environments such that we distinguish other diamagnetic Li-containing species, e.g., LiAlO_2_, from Li_2_CO_3_. For the 10 wt % coating, the signal associated with LNO is very low intensity. This observation suggests that as the diamagnetic component increases, Li is being removed from LNO and supports the hypothesis that Li is able to migrate across the coating–cathode interface during the coating process.

To probe this diamagnetic phase in more detail, *T*_1_ relaxation studies were carried out (Figure S11). For the resonance associated with LNO, saturation recovery experiments estimate *T*_1_ values of 4.3 and 4.9 ms for the 0 and 2 wt % coated samples, respectively. Fast relaxation is expected for a phase containing paramagnetic species. For the 10 wt % coated LNO, the low intensity of the LNO resonance and its overlap with a spinning sideband from the diamagnetic resonance meant that a meaningful *T*_1_ could not be extracted. For the diamagnetic resonances, multiple components are required to fit the relaxation data suggesting the presence of multiple local environments and perhaps overlapping lineshapes. It is possible to fit the *T*_1_ build curve with a variety of multicomponent models; however, this does not always provide a realistic representation of the system. For the 0 wt % Al_2_O_3_ coating on LNO, two components provide a good fit with *T*_1_ relaxation times of 7 ms and 9 s. When compared to that of the 0 wt % coating, the relaxation data for both the 2 and 10 wt % coated samples are fitted best with three components, at least one of which is significantly longer. For the 0 wt % coating, the diamagnetic phases are believed to be present either within the LNO as an impurity or on the surface of LNO. In both these cases, the Li is still in relatively close proximity to the paramagnetic Ni species. The observation of environments with much longer relaxation times could suggest environments further from the paramagnetic center, e.g., Li that has migrated across the coating–cathode interface and into the alumina coating. This is in agreement with the decrease in the intensity of the LNO resonance as the diamagnetic resonance increases in the NMR spectra. Furthermore, this additional diamagnetic environment may correspond to the additional unidentified phase observed in the PXRD data for the 10 wt % coating on LNO.

### 3.4 Effect of Coating Processes on Local Structure

**Figure 7:**
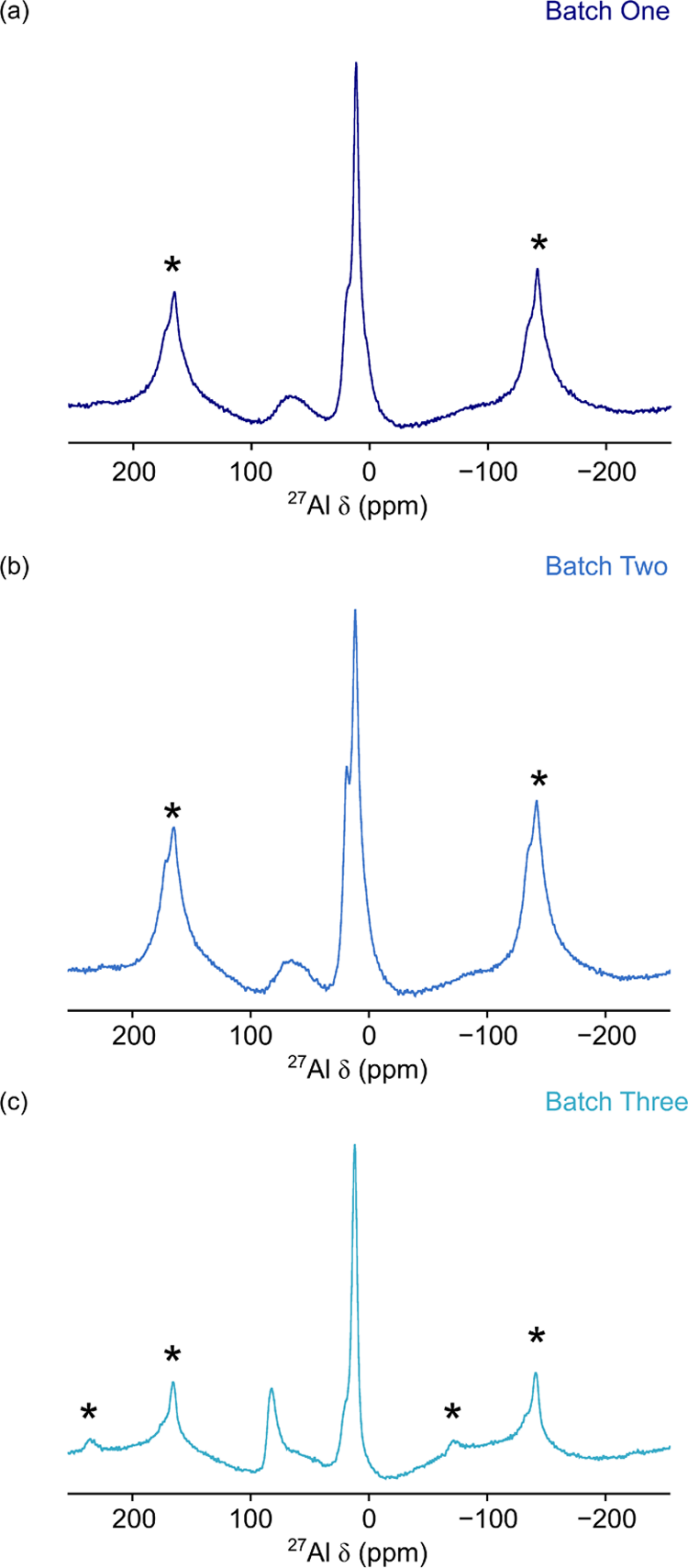
^27^Al MAS NMR spectra for 2 wt % coatings on Al_2_O_3_ on LiNiO_2_ where the same substrate has been coated in three batches. (a–c) Batches one, two, and three, respectively. The spectra are the result of averaging 6400 transients with a recycle interval of 0.5 s. MAS rates of 25 kHz were used, and spinning sidebands are denoted by *.

Previous reports highlight the challenges of reproducibility of coating via wet chemical methods.^41^ This represents a significant challenge when considering industrial scale applications. Either the wet chemical methods must be fully understood and reproducibility improved, or more expensive coating processes must be applied. We examined this further by exposing a sample of coated LNO to air to evaluate the effect of oxygen and moisture on the coating. Figure S12 shows the spectrum acquired for a coating when it had been packed in a glovebox compared with when it had been left in air. No noticeable change was observed. It is worth noting that while no change was observed in the coating via NMR spectroscopy, the electrochemical performance of uncoated LNO is expected to be affected when exposed to air.^42^ Second, a sample of LNO was split into three portions, and each was coated with 2 wt % Al_2_O_3_ following the same procedure. ^27^Al MAS NMR spectra were then acquired (shown in Figure 7). Each spectrum contains resonances corresponding to four- and six-coordinate Al–O species, consistent with previous samples (Figure 5).

The first two spectra acquired (Figure 7a,b) are very similar, with subtle differences observed in the ratio between the overlapping lineshapes in the six-coordinate chemical shift range. This is similar to the differences observed between samples for which the LNO synthetic method was altered (Figure S13). This suggests that the differences are not due to the synthetic method of the LNO but are a consequence of the coating itself, which is consistent with the observation for similar Al environments formed for both LCO and MgO. Furthermore, the spectrum acquired for batch three has significant differences when compared to the other samples. In this spectrum (Figure 7c), an additional narrow resonance is observed in the four-coordinate chemical shift range at δ = 82 ppm. This could possibly be an γ-LiAlO_2_ phase or another similar tetrahedral Al environment. This suggests that the coating process can result in significant variations in the local environment of the coating. However, it is challenging to be more specific about the changes in structure due to the broad nature of the lineshapes. Our work highlights the need for further studies to understand the cause of these structural differences, as they could form during the drying step or during the high-temperature heating.

## 4 Conclusions

With the aim of improving the longevity of nickel-rich cathode materials, the structures of a series of protective alumina coatings have been investigated using solid-state NMR spectroscopy. This greater understanding of the coating’s local structure and the coating–cathode interface will provide valuable insight when improving and optimizing these coatings. Here, a full systematic study (including LiCoO_2_) was particularly important as the structure of the coatings reported in the literature are highly dependent on the coating method.

Both PXRD and TEM data support the successful coating of LNO with an amorphous alumina coating. For the 2 wt % Al_2_O_3_ coating on LNO, a thickness of 6 to 8 nm was measured. Electrochemical characterization shows that although there is a drop in the initial capacity as the result of a 0.2 wt % coating, an improvement in capacity retention of 40% is observed.

The solid-state NMR studies presented here support a disordered structural model composed of four- and six-coordinate Al–O environments. The local Al environments are similar to those seen in other aluminum oxides. The local structure of alumina coatings on LNO has not previously been studied and shows similar structural behavior to coatings on LCO (a diamagnetic analogue).

Without the additional challenges of a paramagnetic substrate, additional information can be obtained for coatings on LCO. In particular, information about the distribution of these environments has been obtained from cross-polarization experiments which suggests that of these environments a subset of the six-coordinate species are in close proximity to protons, likely at the coatings surface. Furthermore, NMR studies also identified that additional phases (LiAlO_2_ and LiCo_1–*x*_Al_*x*_O_2_) are present. These phases are likely formed by the migration of Li and Al across the coating–cathode interface during synthesis. In particular, this is at the elevated temperatures (400 °C) required for forming the coating. Although both these additional phases have previously been identified,^27,28,37,38,43^ the acquisition of high-field NMR data allowed the presence of LiAlO_2_ to be observed for LCO, which had previously been observed for NMC systems.^27^

The presence of these additional phases has important implications for further study of protective coatings. The authors believe that these additional phases are formed at the coating–cathode interface. Thus, instead of a simple coating model, where the Al_2_O_3_ forms a protective shell around the cathode, it may be more accurate to consider a gradual gradient where multiple phases exist within the coating–cathode interface. If this is the case, it should be taken into consideration when designing, tailoring, and improving future coatings.

## Data Availability

Raw data associated with this publication can be accessed at DOI: 10.17635/lancaster/researchdata/653.

## Supporting Information Available

Discussion of computational studies performed (Figures S1–S13) (PDF)

## References

[ref1] Schmuch R.; Wagner R.; Hörpel G.; Placke T.; Winter M. Performance and Cost of Materials for Lithium-based Rechargeable Automotive Batteries. Nat. Energy 2018, 3, 267–278. 10.1038/s41560-018-0107-2.

[ref2] Dunn B.; Kamath H.; Tarascon J.-M. Electrical Energy Storage for the Grid: a Battery of Choices. Science 2011, 334, 928–935. 10.1126/science.1212741. 22096188

[ref3] Wang X.; Ding Y.-L.; Deng Y.-P.; Chen Z. Ni-Rich/Co-Poor Layered Cathode for Automotive Li-Ion Batteries: Promises and Challenges. Adv. Energy Mater. 2020, 10, 1903864 10.1002/aenm.201903864.

[ref4] Booth S. G.; Nedoma A. J.; Anthonisamy N. N.; Baker P. J.; Boston R.; Bronstein H.; Clarke S. J.; Cussen E. J.; Daramalla V.; De Volder M.; Dutton S. E.; Falkowski V.; Fleck N. A.; Geddes H. S.; Gollapally N.; Goodwin A. L.; Griffin J. M.; Haworth A. R.; Haywood M. A.; Hull S.; Inkson B. J.; Johnston B. J.; Lu Z.; MacManus-Driscoll J. L.; Martínez De Irujo Labalde X.; McClelland I.; McCombie K.; Murdock B.; Nayak D.; Park S.; Pérez G. E.; Pickard C. J.; Piper L. F. J.; Playford H. Y.; Price S.; Scanlon D. O.; Stallard J. C.; Tapia-Ruiz N.; West A. R.; Wheatcroft L.; Wilson M.; Zhang L.; Zhi X.; Zhu B.; Cussen S. A. Perspectives for Next Generation Lithium-ion Battery Cathode Materials. APL Mater. 2021, 9, 109201 10.1063/5.0051092.

[ref5] Dahn J. R.; Vonsacken U.; Michal C. A. Structure and Electrochemistry of Li_1±*y*_NiO_2_ and a New Li_2_NiO_2_ Phase with the Ni(OH)_2_ Structure. Solid State Ionics 1990, 44, 87–97. 10.1016/0167-2738(90)90049-W.

[ref6] Dahn J. R.; Vonsacken U.; Juzkow M. W.; Aljanaby H. Rechargeable LiNiO_2_/Carbon Cells. J. Electrochem. Soc. 1991, 138, 2207–2211. 10.1149/1.2085950.

[ref7] Bruce P. G.; Lisowskaoleksiak A.; Saidi M. Y.; Vincent C. A. Vacancy Diffusion in the Intercalation Electrode Li_1–*x*_NiO_2_. Solid State Ionics 1992, 57, 353–358. 10.1016/0167-2738(92)90169-P.

[ref8] Ohzuku T.; Ueda A.; Nagayama M. Electrochemistry and Structural Chemistry of LiNiO_2_ (*R*3*m*) for 4 V Secondary Lithium Cells. J. Electrochem. Soc. 1993, 140, 1862–1870. 10.1149/1.2220730.

[ref9] Bianchini M.; Roca-Ayats M.; Hartmann P.; Brezesinski T.; Janek J. There and Back Again-The Journey of LiNiO_2_ as a Cathode Active Material. Angew. Chem., Int. Ed. 2019, 58, 10434–10458. 10.1002/anie.201812472. 30537189

[ref10] Li C.; Zhang H. P.; Fu L. J.; Liu H.; Wu Y. P.; Rahm E.; Holze R.; Wu H. Q. Cathode Materials Modified by Surface Coating for Lithium Ion Batteries. Electrochem. Acta 2006, 51, 3872–3883. 10.1016/j.electacta.2005.11.015.

[ref11] Kalluri S.; Yoon M.; Jo M.; Liu H. K.; Dou S. X.; Cho J.; Guo Z. Feasibility of Cathode Surface Coating Technology for High-Energy Lithium-ion and Beyond-Lithium-ion Batteries. Adv. Mater. 2017, 29, 1605807 10.1002/adma.201605807. 28251710

[ref12] Kweon H.-J.; Park J. J.; Seo J. W.; Kim G. B.; Jung B. H.; Lim H. S. Effects of Metal Oxide Coatings on the Thermal Stability and Electrical Performance of LiCoCO2 in a Li-ion Cell. J. Power Sources 2004, 126, 156–162. 10.1016/j.jpowsour.2003.08.037.

[ref13] Myung S.-T.; Izumi K.; Komaba S.; Sun Y.-K.; Yashiro H.; Kumagai N. Role of Alumina Coating on Li– Ni– Co– Mn– O Particles as Positive Electrode Material for Lithium-ion Batteries. Chem. Mater. 2005, 17, 3695–3704. 10.1021/cm050566s.

[ref14] Cho J.; Kim Y. J.; Park B. Novel LiCoO2 Cathode Material with Al2O3 Coating for a Li Ion Cell. Chem. Mater. 2000, 12, 3788–3791. 10.1021/cm000511k.

[ref15] Pasqualini M.; Calcaterra S.; Maroni F.; Rezvani S. J.; Di Cicco A.; Alexander S.; Rajantie H.; Tossici R.; Nobili F. Electrochemical and Spectroscopic Characterization of an Alumina-Coated LiMn2O4 Cathode with Enhanced Interfacial Stability. Electrochim. Acta 2017, 258, 175–181. 10.1016/j.electacta.2017.10.115.

[ref16] Oh S.; Lee J. K.; Byun D.; Cho W. I.; Cho B. W. Effect of Al2O3 Coating on Electrochemical Performance of LiCoO2 as Cathode Materials for Secondary Lithium Batteries. J. Power Sources 2004, 132, 249–255. 10.1016/j.jpowsour.2004.01.049.

[ref17] Du K.; Xie H.; Hu G.; Peng Z.; Cao Y.; Yu F. Enhancing the Thermal and Upper Voltage Performance of Ni-Rich Cathode Material by a Homogeneous and Facile Coating Method: Spray-Drying Coating with Nano-Al2O3. ACS Appl. Mater. Interfaces 2016, 8, 17713–17720. 10.1021/acsami.6b05629. 27328728

[ref18] Amaresh S.; Karthikeyan K.; Kim K. J.; Nahm K. S.; Lee Y. S. Alumina Coating on 5 V Lithium Cobalt Fluorophosphate Cathode Material for Lithium Secondary Batteries–Synthesis and Electrochemical Properties. RSC Adv. 2014, 4, 23107–23115. 10.1039/C4RA02318H.

[ref19] Xiang J.; Chang C.; Yuan L.; Sun J. A Simple and Effective Strategy to Synthesize Al2O3-coated LiNi0. 8Co0. 2O2 Cathode Materials for Lithium Ion Battery. Electrochem. Commun. 2008, 10, 1360–1363. 10.1016/j.elecom.2008.07.012.

[ref20] Kosova N.; Devyatkina E.; Slobodyuk A.; Kaichev V. Surface Chemistry Study of LiCoO2 Coated with Alumina. Solid State Ionics 2008, 179, 1745–1749. 10.1016/j.ssi.2008.02.013.

[ref21] Pecher O.; Carretero-González J.; Griffith K. J.; Grey C. P. Materials’ Methods: NMR in Battery Research. Chem. Mater. 2017, 29, 213–242. 10.1021/acs.chemmater.6b03183.

[ref22] Haworth A. R.; Cook C. W.; Griffin J. M.; Solid-State N. M. R. Studies of Coatings and Interfaces in Batteries. Curr. Opin. Colloid Interface Sci. 2022, 62, 101638 10.1016/j.cocis.2022.101638.

[ref23] Mackenzie K. J. D.; Smith M. E. Multinuclear Solid-State Nuclear Magnetic Resonance of Inorganic Materials; Pergamon: Oxford, 2002; Chapter 5.

[ref24] Lee Y.; Woo A. J.; Han K.-S.; Ryu K. S.; Sohn D.; Kim D.; Lee H.; Solid-state N. M. R. Studies of Al-doped and Al2O3-coated LiCoO2. Electrochim. Acta 2004, 50, 491–494. 10.1016/j.electacta.2004.02.063.

[ref25] Fey G. T. K.; Kao H. M.; Muralidharan P.; Kumar T. P.; Cho Y. D. Electrochemical and Solid-State NMR Studies on LiCoO2 Coated with Al2O3 Derived from Carboxylate-alumoxane. J. Power Sources 2006, 163, 135–143. 10.1016/j.jpowsour.2006.01.076.

[ref26] Pol V. G.; Li Y.; Dogan F.; Secor E.; Thackeray M. M.; Abraham D. P. Pulsed Sonication for Alumina Coatings on High-Capacity Oxides: Performance in Lithium-ion Cells. J. Power Sources 2014, 258, 46–53. 10.1016/j.jpowsour.2014.02.030.

[ref27] Han B.; Paulauskas T.; Key B.; Peebles C.; Park J. S.; Klie R. F.; Vaughey J. T.; Dogan F. Understanding the Role of Temperature and Cathode Composition on Interface and Bulk: Optimizing Aluminum Oxide Coatings for Li-ion Cathodes. ACS Appl. Mater. Interfaces 2017, 9, 14769–14778. 10.1021/acsami.7b00595. 28387504

[ref28] Han B.; Key B.; Lapidus S. H.; Garcia J. C.; Iddir H.; Vaughey J. T.; Dogan F. From Coating to Dopant: How the Transition Metal Composition Affects Alumina Coatings on Ni-rich Cathodes. ACS Appl. Mater. Interfaces 2017, 9, 41291–41302. 10.1021/acsami.7b13597. 29091400

[ref29] Riesgo-González V.; Hall D. S.; Märker K.; Slaughter J.; Wright D. S.; Grey C. P. Effect of Annealing on the Structure, Composition, and Electrochemistry of NMC811 Coated with Al2O3 Using an Alkoxide Precursor. Chem. Mater. 2022, 34, 9722–9735. 10.1021/acs.chemmater.2c02580.

[ref30] Brown S. P.; Wimperis S. Two-Dimensional Multiple-Quantum MAS NMR of Quadrupolar Nuclei: A Comparison of Methods. J. Magn. Reson. 1997, 128, 42 10.1006/jmre.1997.1217.

[ref31] Chen Z.; Wang J.; Huang J.; Fu T.; Sun G.; Lai S.; Zhou R.; Li K.; Zhao J. The High-Temperature and High-Humidity Storage Behaviors and Electrochemical Degradation Mechanism of LiNi0.6Co0.2Mn0.2O2 Cathode Material for Lithium Ion Batteries. J. Power Sources 2017, 363, 168–176. 10.1016/j.jpowsour.2017.07.087.

[ref32] Kim Y.; Park H.; Warner J. H.; Manthiram A. Unraveling the Intricacies of Residual Lithium in High-Ni Cathodes for Lithium-ion Batteries. ACS Energy Lett. 2021, 6, 941–948. 10.1021/acsenergylett.1c00086.

[ref33] Lee D.; Duong N. T.; Lafon O.; De Paëpe G. Primostrato Solid-State NMR Enhanced by Dynamic Nuclear Polarization: Pentacoordinated Al3+ Ions Are Only Located at the Surface of Hydrated γ-Alumina. J. Phys. Chem. C 2014, 118, 15065–25076. 10.1021/jp508009x.

[ref34] Morris H. D.; Ellis P. D. Aluminum-27 Cross Polarization of Aluminas. The NMR spectroscopy of Surface Aluminum Atoms. J. Am. Chem. Soc. 1989, 111, 6045–6049. 10.1021/ja00198a012.

[ref35] Barrow N. S.; Scullard A.; Collis N. Surface Selective 1H and 27Al MAS NMR Observations of Strontium Oxide Doped γ-alumina. Johnson Matthey Technol. Rev. 2016, 60, 90–97. 10.1595/205651316X690943.

[ref36] Shkrob I. A.; Gilbert J. A.; Phillips P. J.; Klie R.; Haasch R. T.; Bareño J.; Abraham D. P. Chemical Weathering of Layered Ni-rich Oxide Electrode Materials: Evidence for Cation Exchange. J. Electrochem. Soc. 2017, 164, A1489 10.1149/2.0861707jes.

[ref37] Han B.; Key B.; Lipton A. S.; Vaughey J. T.; Hughes B.; Trevey J.; Dogan F. Influence of Coating Protocols on Alumina-Coated Cathode Material: Atomic Layer Deposition versus Wet-Chemical Coating. J. Electrochem. Soc. 2019, 166, A3679–A3684. 10.1149/2.0681915jes.

[ref38] Han B.; Dunlop A. R.; Trask S. E.; Key B.; Vaughey J. T.; Dogan F. J. Tailoring Alumina Based Interphases on Lithium Ion Cathodes. Electrochem. Soc. 2018, 165, A3275–A3283. 10.1149/2.0211814jes.

[ref39] Gaudin E.; Taulelle F.; Stoyanova R.; Zhecheva E.; Alcántara E.; Lavela P.; Tirado J. L. Cobalt(III) Effect on 27Al NMR Chemical Shifts in LiAlxCo1-xO2. J. Phys. Chem. B 2001, 105, 8081–8087. 10.1021/jp0105948.

[ref40] Li H.; Hua W.; Liu-Théato X.; Fu Q.; Desmau M.; Missyul A.; Knapp M.; Ehrenberg H.; Indris S. New Insights into Lithium Hopping and Ordering in LiNiO2 Cathodes during Li (De)intercalation. Chem. Mater. 2021, 33, 9546–9559. 10.1021/acs.chemmater.1c02680.

[ref41] Nisar U.; Muralidharan N.; Essehli R.; Amin R.; Belharouak I. Valuation of Surface Coatings in High-Energy Density Lithium-ion Battery Cathode Materials. Energy Storage Mater. 2021, 38, 309–328. 10.1016/j.ensm.2021.03.015.

[ref42] Mu L.; Yang Z.; Tao L.; Waters C. K.; Xu Z.; Li L.; Sainio S.; Du Y.; Xin H. L.; Nordlundd D.; Lin F. The Sensitive Surface Chemistry of Co-Free, Ni-Rich Layered Oxides: Identifying Experimental Conditions That Influence Characterization Results. J. Mater. Chem. A 2020, 8, 17487–17497. 10.1039/D0TA06375D.

[ref43] Negi R. S.; Celik E.; Pan R.; Stäglich R.; Senker J.; Elm M. T. Insights into the Positive Effect of Post-Annealing on the Electrochemical Performance of Al2O3-Coated Ni-Rich NCM Cathodes for Lithium-Ion Batteries. ACS Appl. Energy Mater. 2021, 4, 3369–3380. 10.1021/acsaem.0c03135.

