# Supplementary material for: Structural Insight into Protective Alumina Coatings for Layered Li-Ion Cathode Materials by Solid-State NMR Spectroscopy
Source: ACS Appl Mater Interfaces. 2024 Feb 2;16(6):7171–81. doi: 10.1021/acsami.3c16621 (PMC10875645; doi:10.1021/acsami.3c16621)
Supplement: Supplementary file 1 — am3c16621_si_001.pdf [file am3c16621_si_001.pdf]

Supporting Information

Structural Insight into Protective Alumina Coatings for Layered Li-ion Cathode Materials by Solid-State NMR Spectroscopy

Abby R. Haworth,[1,5,†] Beth I. J. Johnston,[2,5] Laura Wheatcroft,[2,5,§] Sarah L. McKinney,[1,3,5] Nuria Tapia-Ruiz,[3,5] Sam G. Booth,[2,5,‡] Alisyn J. Nedoma,[4,5] Serena A. Cussen,[2,5] and John M. Griffin[1,5]*

1 Department of Chemistry, Lancaster University, Lancaster, LA1 4YB, UK

2 Department of Materials Science and Engineering, University of Sheffield, Sheffield S1 3JD, UK

3 Department of Chemistry, Molecular Sciences Research Hub, White City Campus, Imperial College London, London W12 0BZ, UK

4 Department of Chemical and Biological Engineering, University of Sheffield, Sheffield, S1 3JD, UK

5 The Faraday Institution, Quad One, Harwell Campus, OX11 0RA, UK

Email j.griffin@lancaster.ac.uk

Present Addresses

† Department of Materials, Design, and Manufacturing Engineering, School of Engineering, University of Liverpool, Liverpool, L69 3GH, UK

§ Nyobolt Ltd. Evolution Business Park, Cambridge, CB24 9NG, UK

‡ Integrated Graphene Ltd. Euro House, Stirling, FK8 2DJ, UK

Table of Contents:

Figure S1: Cycling profile for 0.2 wt% coating of Al_2O_3 on $LiNiO_2$	Page 2
Figure S2: PXRD pattern for unprocessed $LiCoO_2$ as well as 0, 0.2, 2, and 10 wt% coatings of Al_2O_3 on $LiCoO_2$	Page 2
Figure S3: The ^{27}Al MQMAS NMR spectrum of a 10 wt% Al_2O_3 coating on $LiCoO_2$	Page 3
Figure S4: The ^{27}Al MAS NMR spectrum of Al_2O_3 synthesised via the coating method	Page 3
Figure S5: The ^{27}Al MAS NMR spectra of 10 and 0.2 wt% coatings of Al_2O_3 on MgO	Page 4
Figure S6: The ^{27}Al MAS NMR spectra of 10 and 0.2 wt% coatings of Al_2O_3 on $LiCoO_2$ acquired at 16.4 T	Page 5
Figure S7: The ^{27}Al MAS NMR spectra of $LiAlO_2$ and Al_2O_3 alongside PXRD pattern of $LiAlO_2$	Page 6
Table S1: The calculated chemical shielding and chemical shift for a number of Li and Al containing phases	Page 7
Figure S8: The ^{27} Al MAS NMR spectrum of a 0.2 wt% Al_2O_3 coating on $LiCoO_2$ acquired using a Hahn Echo	Page 8
Figure S9: The ^{27}Al MAS NMR spectrum of $LiNi_{0.95}Al_{0.05}O_2$	Page 8
Figure S10: The ^{27}Al MAS NMR spectra of a 0.2 wt% Al_2O_3 coating on $LiNiO_2$	Page 9
Figure S11: T_1 relaxation curves for 0, 2, and 10 wt% coatings of Al_2O_3 on $LiNiO_2$	Page 10
Figure S12: The ^{27}Al MAS NMR spectrum for 10 wt% Al_2O_3 coatings on $LiNiO_2$ left in air	Page 11
Figure S13: The ^{27}Al MAS NMR spectra for 2 wt% Al_2O_3 coatings on $LiNiO_2$ for two different methods of $LiNiO_2$ synthesis	Page 11
References	Page 12

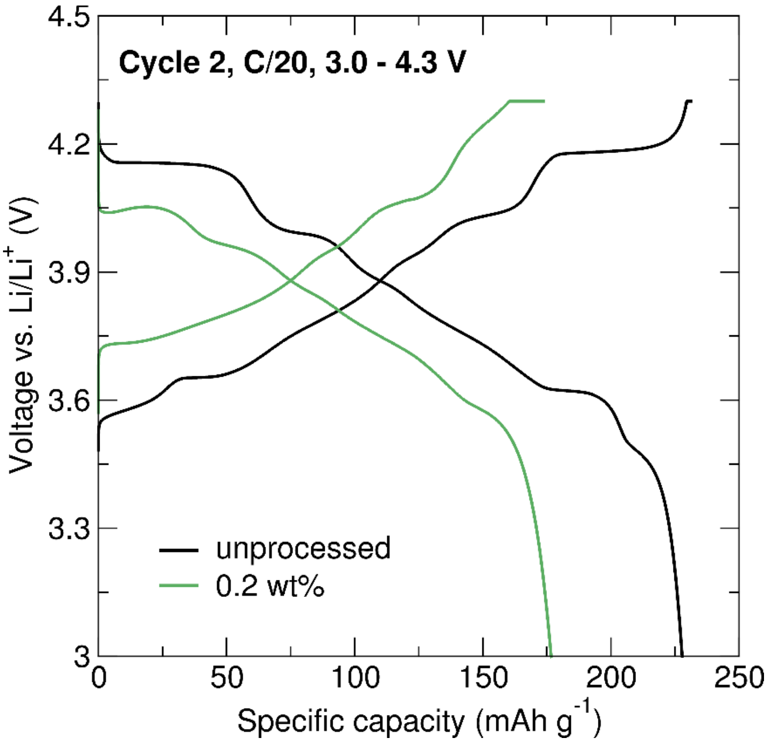

Figure S1: Voltage-capacity profiles recorded during the 2nd cycle for uncoated $LiNiO_2$ and 0.2 wt% coating of Al_2O_3 on $LiNiO_2$. Half cells were cycled between 3.0 to 4.3 V vs. Li/Li^+ at a rate of C/20.

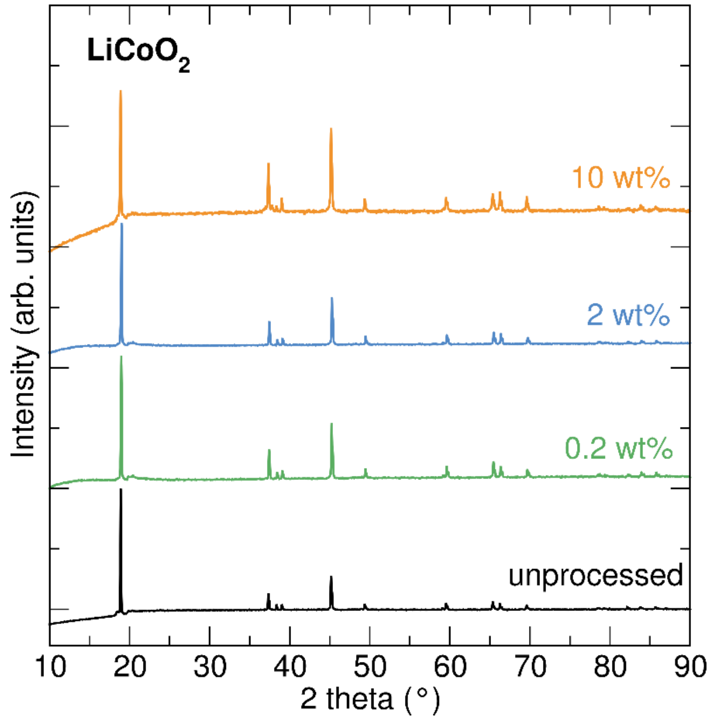

Figure S2: Powder X-ray diffraction patterns for unprocessed $LiCoO_2$ and 0, 0.2, 2, and 10 wt% coating of Al_2O_3 on $LiCoO_2$.

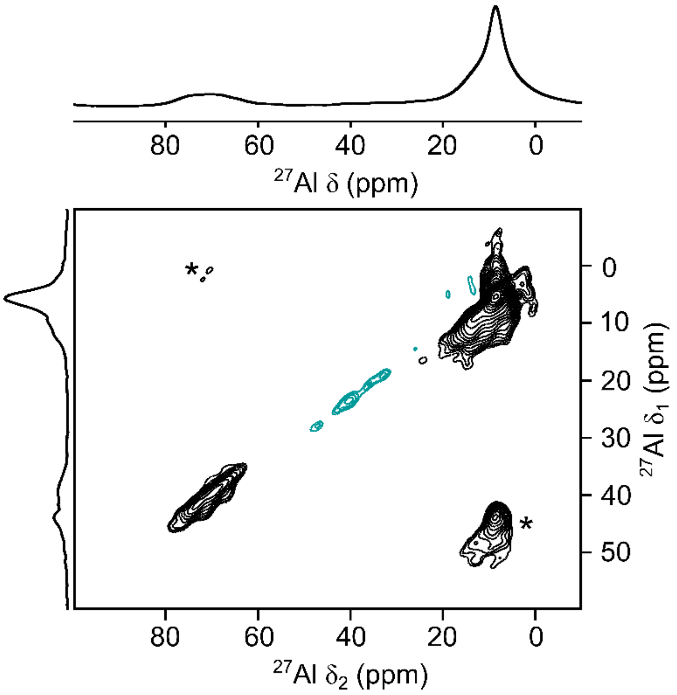

Figure S3: The ^{27}Al MQMAS NMR (16.4 T) spectrum of 10 wt% Al_2O_3 coating on $LiCoO_2$. This MQMAS NMR spectrum is the result of averaging 9600 transients with a recycle interval of 0.5 s for 30 intervals of 71.76 μs. A MAS rate of 18 kHz was used and spinning sidebands are denoted by *.

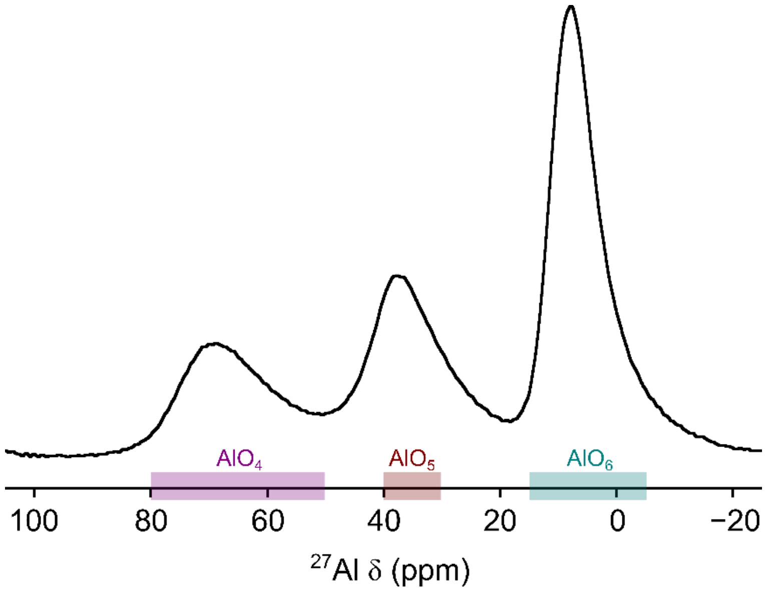

Figure S4: ^{27}Al MAS NMR (16.4 T) spectrum of Al_2O_3 synthesised via the coating method. The spectrum is the result of averaging 64 transients with a recycle delay of 0.5 s. A MAS rate of 18 kHz was employed and spinning sidebands are denoted with *. The pink, red, and green boxes represent the chemical shift range report in literature for four-, five-, and six-coordinate Al-O environments, respectively.

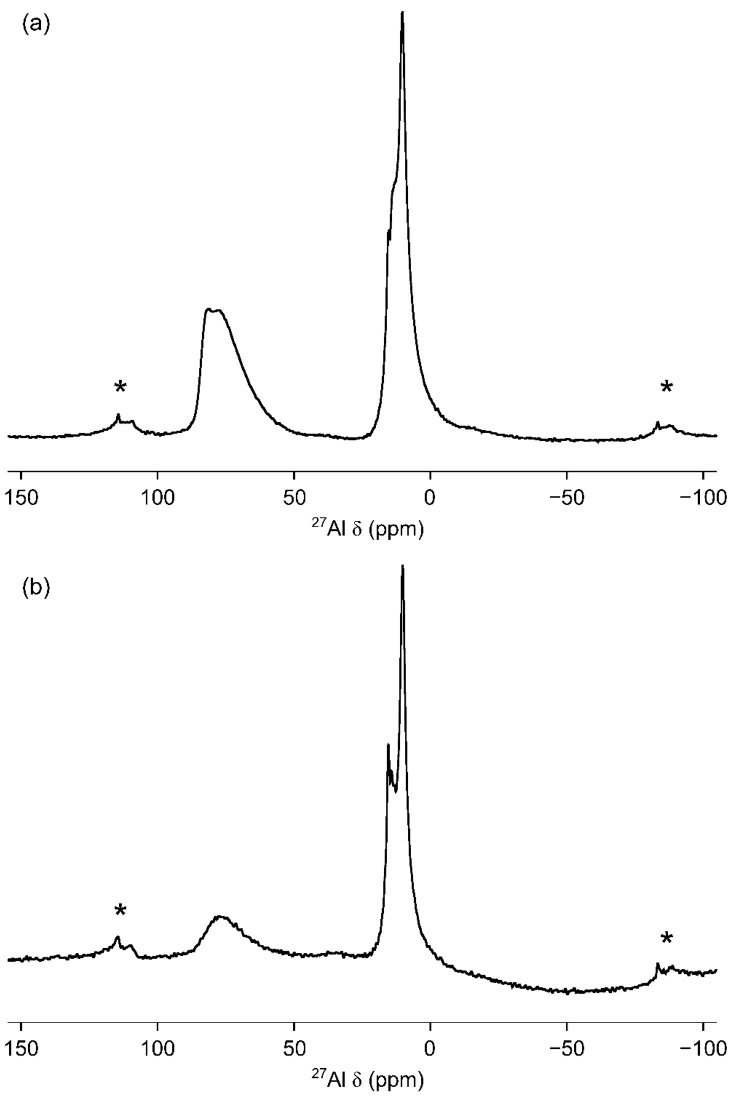

Figure S5: ^{27}Al MAS NMR (16.4 T) Spectra for (a) 10 wt% and (b) 0.2 wt% coatings of Al_2O_3 on MgO. Each spectrum is the result of averaging (a) 6400 and (b) 196144 transients with a recycle interval of 0.5 s. MAS rates of 18 kHz were used and spinning sidebands are denoted with *.

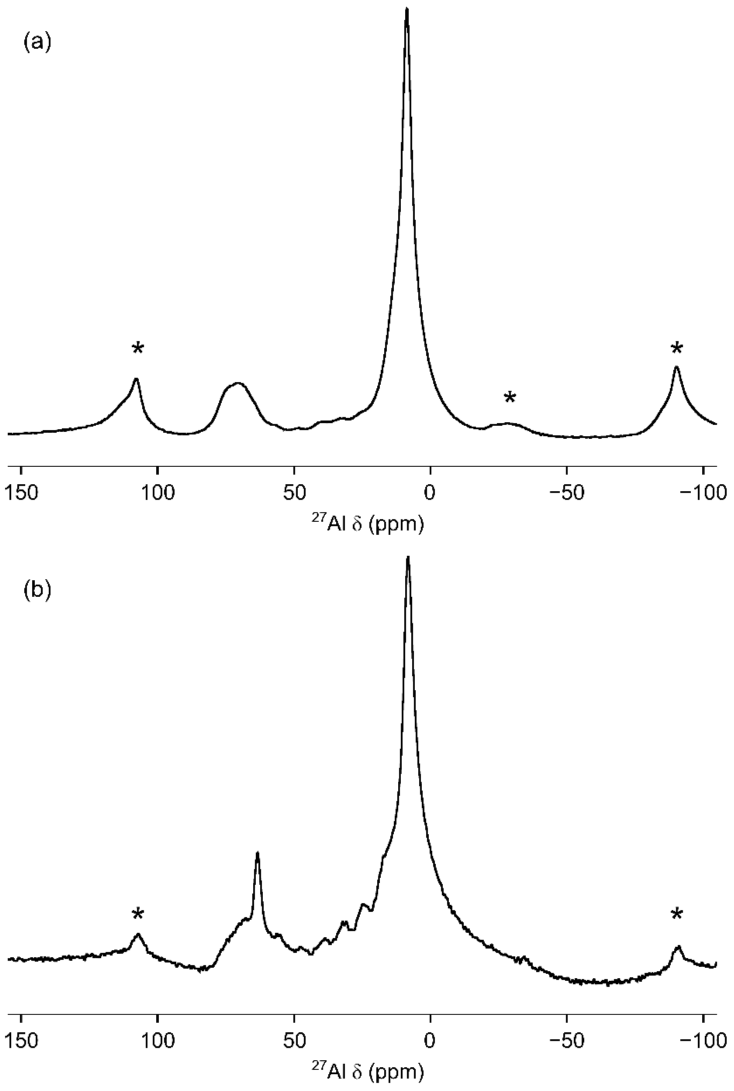

Figure S6: ^{27}Al MAS NMR (16.4 T) spectra for (a) 10 wt% and (b) 0.2 wt% Al_2O_3 coatings on $LiCoO_2$. Each spectrum is the result of averaging (a) 16000 and (b) 149872 transients with a recycle interval of 0.5 s. MAS rates of 18 kHz were employed and spinning sidebands are denoted with *.

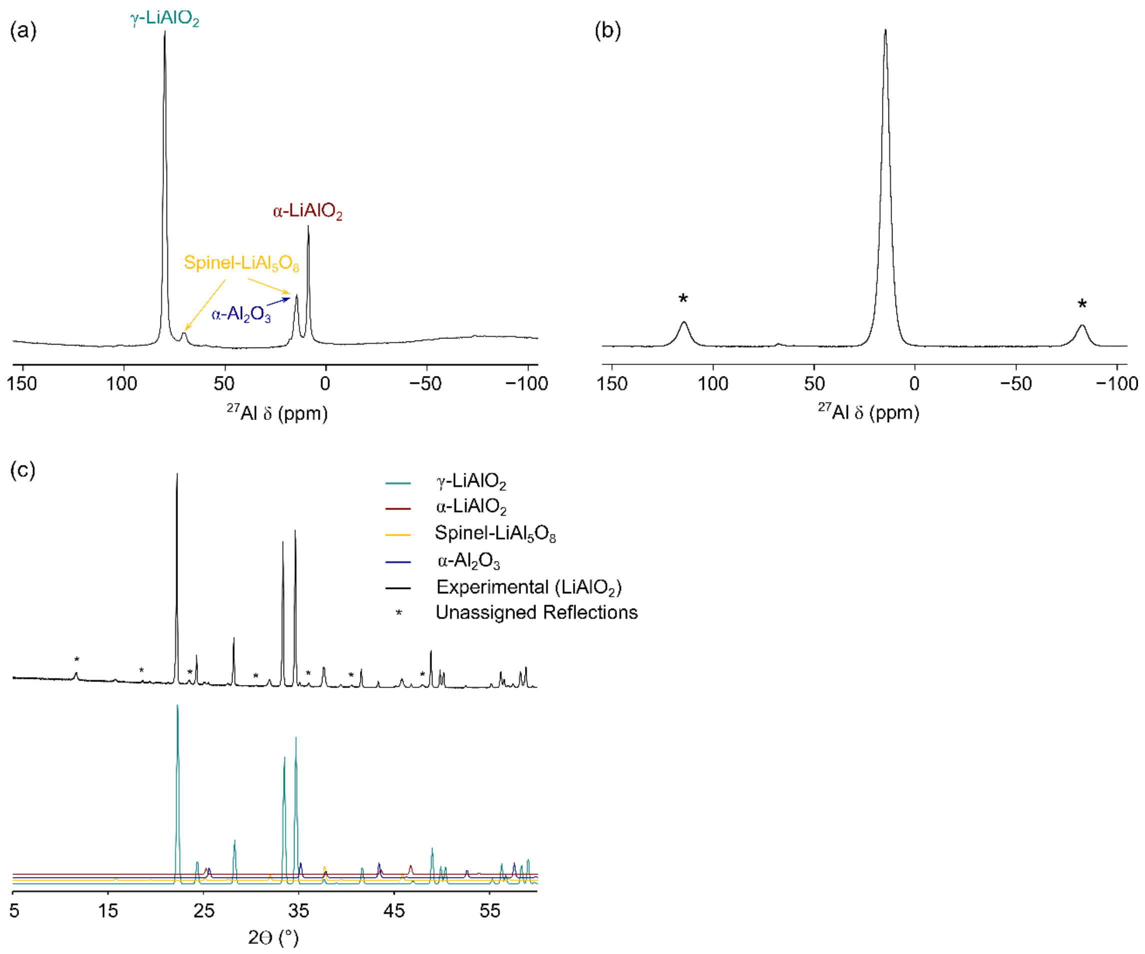

Figure S7: ^{27}Al MAS NMR spectrum of (a) $LiAlO_2$ (Alfa Aesar) acquired at 23.5 T and (b) Al_2O_3 (ACROS – 99.7%) acquired at 16.4 T. The spectrum is the result of averaging (a) 320 and (b) 64 with a recycle interval of 0.5 s. MAS rates of (a) 40 and (b) 18 kHz were used and spinning sidebands are denoted with *. (c) shows the powder X-ray diffraction (PXRD) pattern acquired for $LiAlO_2$ (Alfa Aesar) and patterns predicted for α-Al_2O_3, α-$LiAlO_2$, γ-$LiAlO_2$, and spinel-$LiAl_5O_8$, for comparison. In (c), * denotes unassigned reflections.

The $LiAlO_2$ obtained from Alfa Aesear was made up of multiple phases and PXRD and density function theory (DFT) calculations were used to help assign the multiple resonances observed in the ^{27}Al MAS NMR spectrum (Figure S7 (a)). The PXRD data suggests the presence of α-Al_2O_3, α-$LiAlO_2$, γ-$LiAlO_2$, and spinel-$LiAl_5O_8$ in addition to additional unassigned phase(s). To aid in assigned the resonances resulting from additional phases, the values for the isotropic chemical shielding (σ_{iso}) for these phases were obtained from DFT calculations (shown in the Table S1). Chemical shift values (δ_{iso}) can then be obtained by referencing these to a known systems, *e.g.*, Al_2O_3 and $Li(PO_3)_4$. However, it is noted that δ_{iso} values were reference dependant (Table S1). Calibration using Al_2O_3 provides δ_{iso} values closer to what is observed in literature for phases such as γ-$LiAlO_2$ (δ = 76 ppm[1] to 80 ppm[2]). This is likely due to it being a similar Al-O system. Thus, based on this, the resonance at δ = 80 ppm is assigned to γ-$LiAlO_2$, the resonances at δ = 71 and 14 ppm are assigned to the spinel $LiAl_5O_8$ phase. It is noted that the resonance at δ = 14 ppm may also be

due to Al_2O_3 based on comparison of Figures S7 (a) with (b). The resonance at δ = 9 ppm is assigned to α-$LiAlO_2$.

Table S1: The chemical shielding (σ_{iso}) calculated for a number of phases using DFT calculations and the corresponding chemical shifts (δ_{iso}) calculated using $Al(PO_3)_4$ and Al_2O_3 as a reference.

Phase	Site	σ_{iso} (ppm)	δ_{iso} (ppm) based on calibration to $AlPO_4$ (chabazite)	δ_{iso} (ppm) based on calibration to Al_2O_3
α-$LiAlO_2$	Al1	542.31	20.2	12.2
Al_2O_3	Al1	540.57	21.9	14.0
γ-$LiAlO_2$	Al1	476.73	85.7	77.9
Spinel-$LiAl_5O_8$	Al1	485.76	76.7	68.8
Spinel-$LiAl_5O_8$	Al2	540.89	21.6	13.7
$AlPO_4$ (chabazite)	Al1	523.06	39.4 (known)	31.5

Experimental Methods:

First-principles calculations of NMR parameters were carried out using the CASTEP code[3] employing the gauge-including projector augmented wave (GIPAW) algorithm,[4] which allows the reconstruction of the all-electron wave function in the presence of a magnetic field. The CASTEP calculations employed the generalised gradient approximation Perdew–Burke–Ernzerhof exchange-correlation functional,[5] and core–valence interactions were described by ultrasoft pseudopotentials.[6]

Prior to calculation of the NMR parameters, structures were fully geometry optimised, allowing all atomic positions and unit cell parameters to vary. Geometry optimisations and NMR calculations were carried out using a planewave energy cut-off of 800 eV and a k-point spacing of 0.03 2π $Å^{-1}$ was used. The calculations generate the absolute shielding tensor (σ) in the crystal frame. Diagonalisation of the symmetric part of σ yields the three principal components, σ_{XX}, σ_{YY}, and σ_{ZZ}. The isotropic shielding, σ_{iso}, is given by (1/3) Tr [σ]. The isotropic chemical shift, δ_{iso}, is given by $\sigma_{ref} - \sigma_{iso}$, where σ_{ref} is a reference shielding.

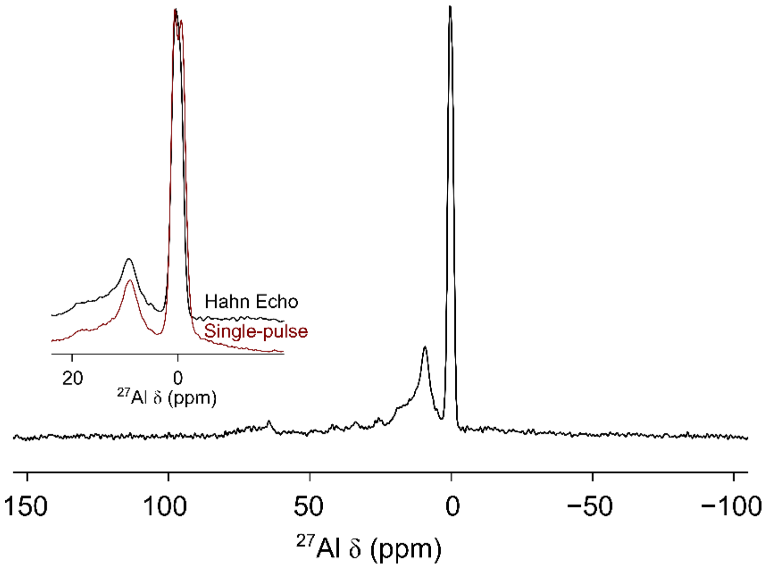

Figure S8: ^{27}Al MAS NMR (23.5 T) spectrum of 0.2 wt% coating on Al_2O_3 on $LiCoO_2$ acquired via a Hahn echo experiment (90 – τ – 180 – τ). The spectrum is the result of averaging 42,400 transients with a recycle delay of 0.5 s. Pulse lengths of 2.75 and 30 μs were used for the 90 and low power 180 pulses, respectively. A MAS rate of 40 kHz was used and spinning sidebands are denoted with *. An overlay with the spectrum acquired via a conventional single-pulse experiment is shown in the inset.

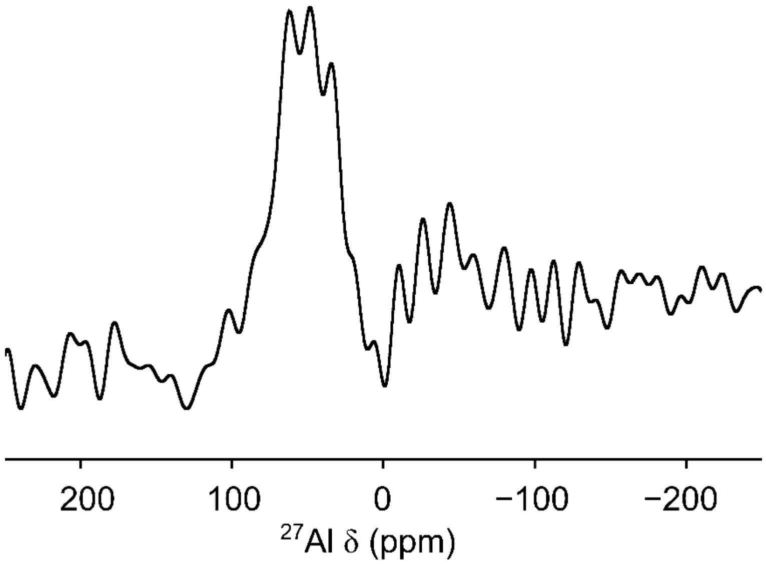

Figure S9: ^{27}Al MAS NMR (23.5 T) spectrum of $LiNi_{0.95}Al_{0.05}O_2$. The spectrum is the result of averaging 2176 transients with a recycle interval of 0.5 s. A MAS rate of 40 kHz was used.

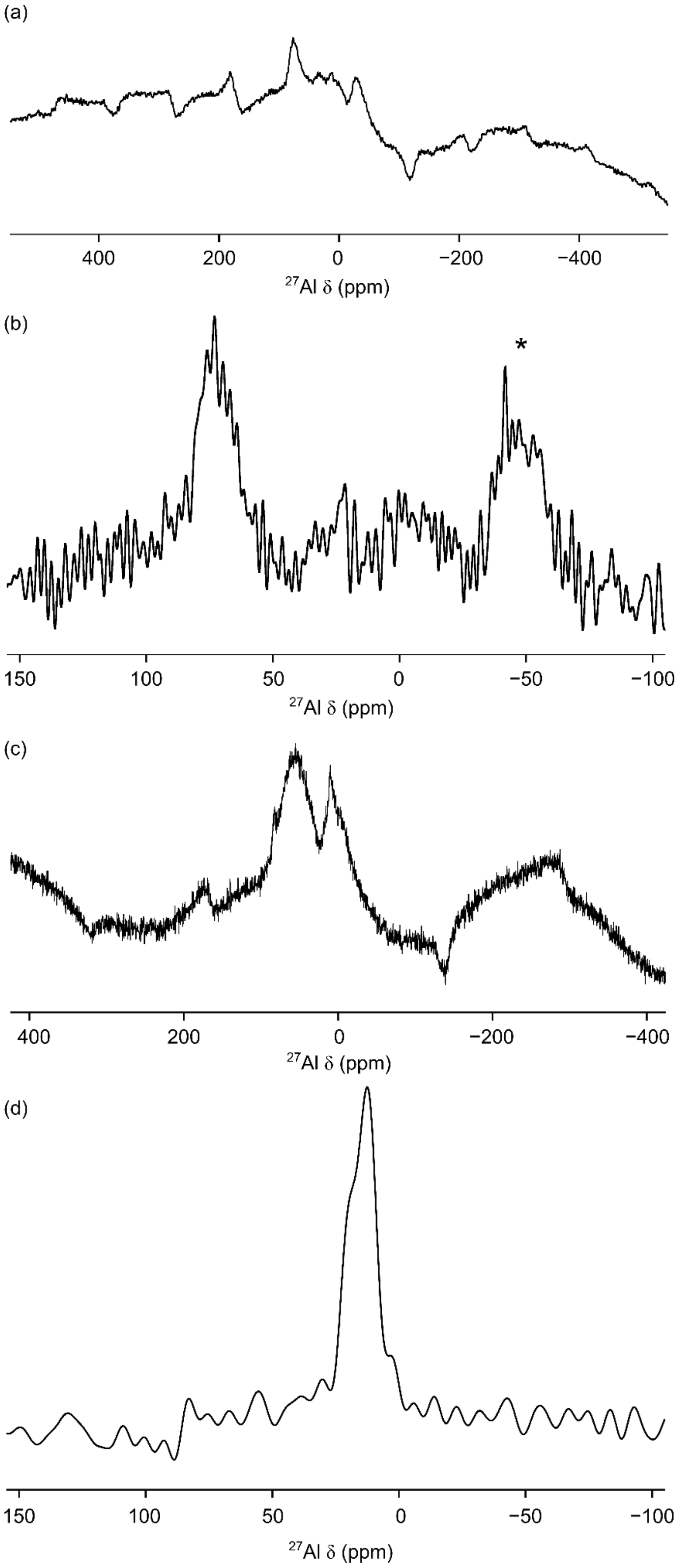

Figure S10: ^{27}Al MAS NMR spectrum of 0.2 wt% Al_2O_3 coating on $LiNiO_2$ (a) at 16.4 T, (b) at 16.4 T with a Hahn Echo experiment, (c) at 23.5 T, and (d) at 23.5 T with a Hahn Echo experiment. These spectra are the result of averaging (a) 168000, (b) 168000, (c) 29696 and (d) 53360 transients with a recycle interval of 0.5 s. MAS rates of (a) and (b) 18 kHz or (c) and (d) 40 kHz were used.

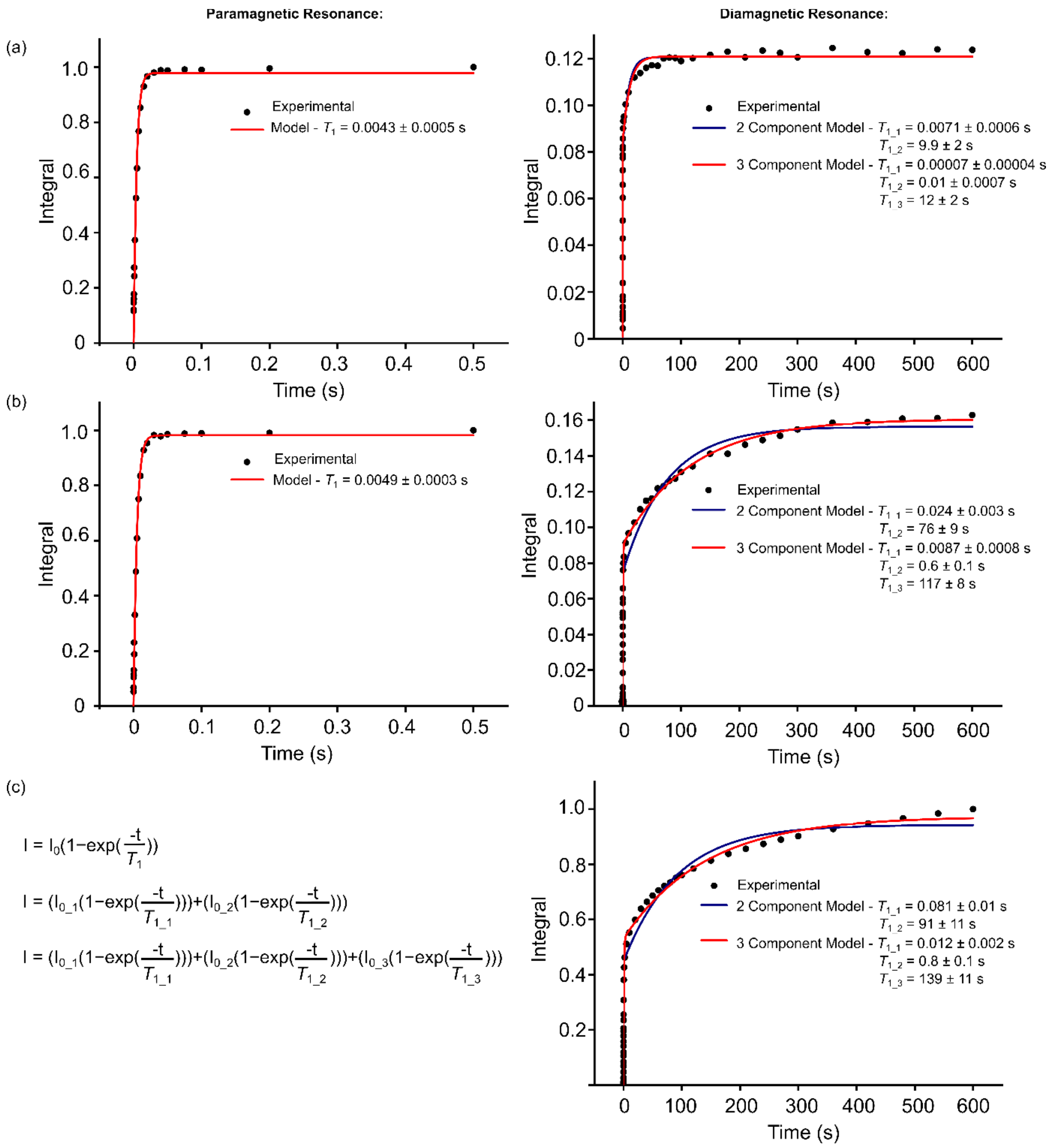

Figure S11: T_1 relaxation curves extracted from saturation recovery experiments for (a) 0 wt%, (b) 2 wt%, and (c) 10 wt% coatings of Al_2O_3 on $LiNiO_2$. The data is fitted with either one, two, or three components.

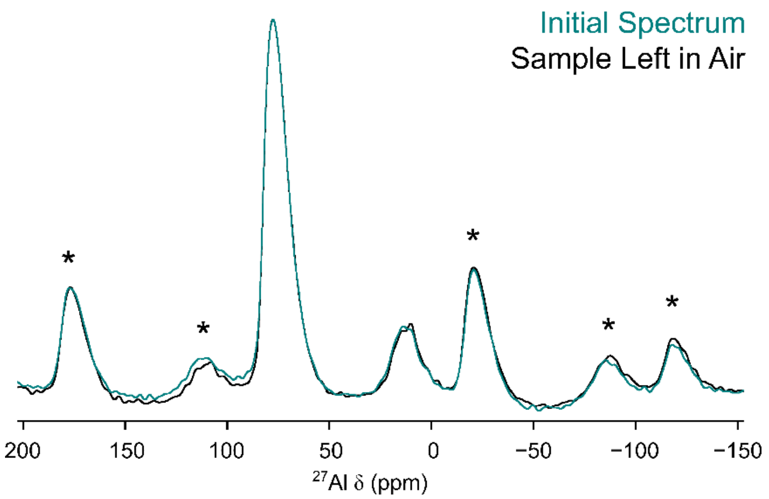

Figure S12: The ^{27}Al MAS NMR (23.5 T) spectra for a 10 wt% coating of Al_2O_3 on $LiNiO_2$ before and after it had been left in air. These spectra are the result of averaging transients with a recycle interval of 0.5 s. MAS rates of 18 kHz were employed and spinning sidebands are denoted by *

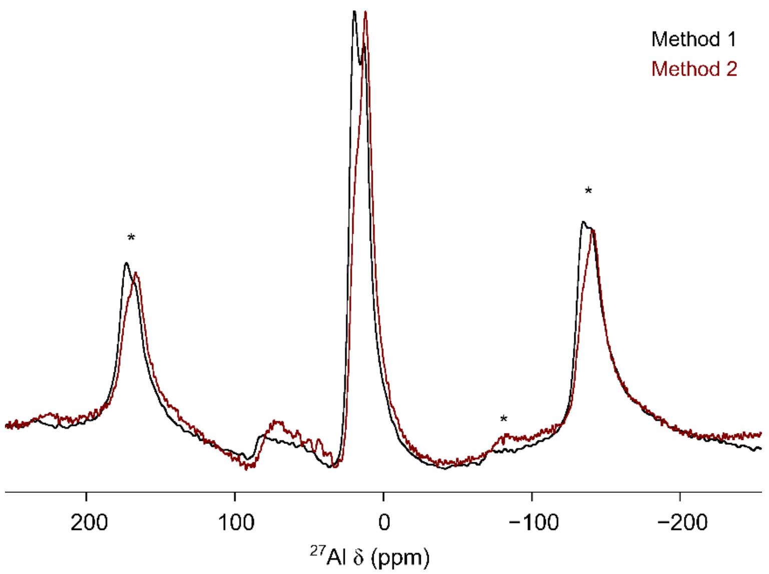

Figure S13: The ^{27}Al MAS NMR (23.5 T) spectra for 2 wt% coating of Al_2O_3 on $LiNiO_2$ where $LiNiO_2$ is synthesised via method 1 and method 2. These spectra are the result of averaging 6400 transients with a recycle interval of 0.5 s. MAS rates of 40 kHz are used and spinning side bands are denoted with a *.

References:

1. B. Han, T. Paulauskas, B. Key, C. Peebles, J. S. Park, R. F. Klie, J. T. Vaughey, and F. Dogan, *ACS Appl. Mater. Interfaces* 2017, **9**, 14769-14778.
2. E. Gaudin, F. Taulelle, R. Stoyanova, E. Zhecheva, E. Alcántara, P. Lavela, and J. L. Tirado, *J. Phys. Chem. B* 2001, **105**, 8081-8087.
3. M. D. Segall, P. J. D. Lindan, M. J. Probert, C. J. Pickard, P. J. Hasnip, S. J. Clark, M. C. Payne, *J. Phys. Condens. Matter,* 2002, **14**, 2717-2744.
4. C. J. Pickard, F. Mauri, *Phys. Rev. B*, 2001, **63**, 245101.
5. J. P. Perdew, K. Burke, M. Ernzerhof, *Phys. Rev. Lett.* 1996, **77**, 3865-3868.
6. J. R. Yates, C. J. Pickard, F. Mauri, *Phys. Rev. B*, 2007, **76**, 24401.
